# Multi-targeted MS-based metabolomics fingerprinting of black and white pepper coupled with molecular networking in relation to their *in vitro* antioxidant and antidiabetic effects

**DOI:** 10.1039/d5ra03714j

**Published:** 2025-08-04

**Authors:** Mostafa H. Baky, Amal A. Maamoun, Alexandru Nicolescu, Andrei Mocan, Mohamed A. Farag

**Affiliations:** a Department of Pharmacognosy, Faculty of Pharmacy, Egyptian Russian University Badr City Cairo 11829 Egypt; b Pharmacognosy Department, National Research Centre 33 El Buhouth Street, Dokki, P. O. 12622 Giza Egypt; c Laboratory of Chromatography, Institute of Advanced Horticulture Research of Transylvania, University of Agricultural Sciences and Veterinary Medicine 3-5 Mănăştur Street 400372 Cluj-Napoca Romania; d Department of Pharmaceutical Botany, “Iuliu Hațieganu” University of Medicine and Pharmacy Gheorghe Marinescu Street 23 Cluj-Napoca 400337 Romania; e Pharmacognosy Department, College of Pharmacy, Cairo University 11562 Cairo Egypt mohamed.farag@pharma.cu.edu.eg

## Abstract

Spices are considered as a valuable food material owing not only to their special aroma, but also a myriad of nutritional and health benefits. Black pepper (*Piper nigrum* L.; Piperaceae) is known as the “king of spices”, being commonly used worldwide in its two forms: black and processed white pepper. The main goal of this study was to perform multi-targeted comparative metabolite profiling and fingerprinting approaches targeting primary and secondary metabolites using gas chromatography mass-spectrometry (GC-MS) post-silylation and ultra-performance liquid chromatography (UPLC-MS/MS) coupled to multivariate analyses and molecular networking. A total of 51 metabolites were annotated using GC-MS belonging to fatty acids/esters (9), alkaloids/nitrogenous (6), sugars (3), sugar alcohols (5), organic acids (15), alcohols (4), and aliphatic hydrocarbons (6) in addition to phenols (3). Fatty acids/esters were enriched in black and white pepper at *ca.* 23.4 mg g^−1^. Moreover, piperine was detected at higher levels in white pepper at 5.9 mg g^−1^ compared to 3.4 mg g^−1^ in black pepper. A total of 71 metabolites were annotated using UPLC-MS/MS, with piperamides as the most abundant class, of which 6 are first time to be detected in *P. nigrum* fruit “types A, E and O”. In addition, 7 fatty acids were recoded along 4 flavonoids exhibiting novel glycosidic linkage of kaempferol and apigenin. Furthermore, 5 hydroxycinnamic acids have been detected; some were identified for the first time from *P. nigrum* fruit. Clusters of fatty acids, flavonoids and phenylamides were detected by negative mode GNPS molecular networking, whereas clusters representing the majority of alkaloids were detected in positive mode. Assay of total phenolics and flavonoids revealed higher levels in black compared to white pepper, with values of 45.6 and 37.5 mg GAE per g for total phenolics and 9.4 & 8.5 mg RE per g for flavonoids, respectively. Assessment of antioxidant capacity using DPPH, ABTS scavenging assays, and FRAP assay revealed moderate effects at 49.79, 20.6, and 104.6 (black pepper), 29.0, 11.5, and 77.5 mg TE per g (white pepper), respectively. Moreover, black and white pepper extracts inhibited α-glucosidase enzyme with an IC_50_ of 0.77 and 0.62 mg mL^−1^, compared with acarbose.

## Abstract 2

Spices are considered as a valuable food material owing not only to their special aroma, but also a myriad of nutritional and health benefits.

## Graphical abstract

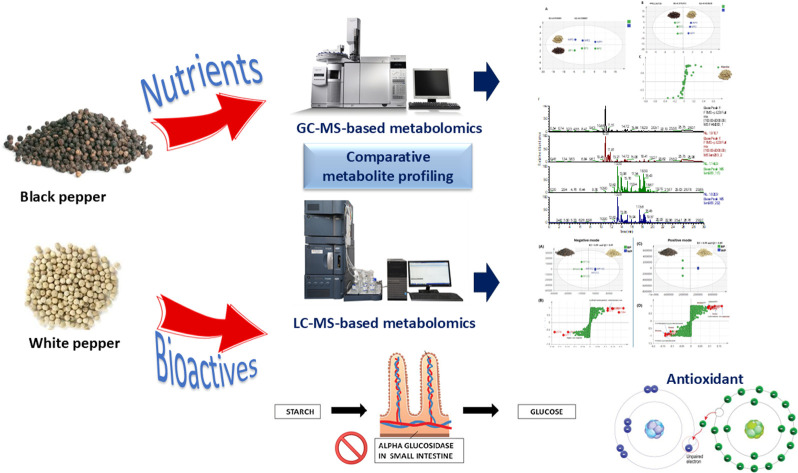

## Introduction

Spices are considered as one of the chief sources for food additives with potential health benefits owing to not only their characteristic aroma but their richness in bioactive secondary metabolites.^1^ Spices are a rich source of biologically active compound classes including flavonoids, carotenoids, terpenes and others.^2^ Asides from their culinary uses, they exert a pivotal role in human health, exemplified by carminative, analgesic, stomachic, antiviral, anti-inflammatory, and antioxidant effects.^3^ Among the most important spices, black pepper (*Piper nigrum* L.; Piperaceae) and its processed variant, white pepper (produced by peeling of the outer black layer) are widely consumed worldwide.^4^

*Piper nigrum* is enriched with wide groups of bioactive secondary metabolites such as alkaloids (notably piperine), essential oil, lignans, phenolics, carotenoids, and terpenoids, with potential health value. *P. nigrum* is widely used in traditional medicine for the treatment of gastric complaints cough, cold, and intermittent fever, as well as being a rubefacient, stimulant, appetite stimulant, and anti-inflammatory product.^5^ The characteristic aroma and pungency of black pepper are attributed to piperine and essential oils.^5^ Piperine is the major bioactive alkaloid in black pepper, and it exhibits various therapeutic effects, including antihypertensive, anticancer, antioxidant, analgesic, and antidepressant.^5^ Black pepper is used to enhance the bioavailability of several drugs *via* inhibition of several digestive enzymes, *i.e.*, alpha-glucosidase, leading to improved therapeutic value.^6^ Aside from alkaloids, essential oils and flavonoids constitute the major phytonutrients in black pepper.

Metabolites profiling in spices is important to unveil their chemical composition and ensure their medicinal value. Nowadays, both targeted and untargeted metabolomics approaches are increasingly reported for profiling different spices to ensure their quality.^3^ Owing to its high sensitivity, gas chromatography mass spectrometry technique (GC-MS) especially after derivatization is suited for profiling of both aroma and nutrient low molecular weight metabolites, *i.e.*, sugars and organic acids.^7^ In contrast, ultra-high-performance liquid chromatography coupled with high-resolution tandem mass spectrometry (UHPLC-HRMS/MS) is well-suited for profiling thermolabile and non-volatile secondary metabolites that are often linked to health-promoting effects. Each analytical platform offers distinct advantages, as GC-MS provides excellent separation and sensitivity for small metabolites, while UPLC-HRMS/MS allows access to a broader range of complex phytochemicals. To manage and interpret the complexity of the resulting metabolomic datasets, multivariate statistical analyses are employed. These include unsupervised tools such as principal component analysis (PCA), hierarchical cluster analysis (HCA), as well as supervised techniques like orthogonal projections to latent structures discriminant analysis (OPLS-DA), which facilitate sample classification and the identification of discriminative metabolite markers.^8^

Diabetes mellitus has been recognized as one of the most crippling diseases with huge social, health, and economic consequences.^9^ As the final step in carbohydrate metabolism is mediated by α-glucosidase in the brush border of the enterocytes.^10^ The inhibition of the activity of this enzyme is considered one of the applied strategies to control blood glucose levels.^11^ Recently, considerable interest has been paid to the antioxidant impact of naturally occurring phytochemicals in foods and spices for the human body.^12^ Owing to different mechanistic pathways for the antioxidant capacity of plant-based phytochemicals, several assays were displayed to evaluate their potential in reducing oxidative stress-related diseases, including DPPH and ABTS scavenging effect.^12^

This study aims to extend upon our previous work by providing a more comprehensive and comparative analysis of the metabolite heterogeneity between black and white pepper. While our previous study utilized SPME-GC-MS and NMR-based metabolomics targeting sensory and nutritive determinants,^13^ the current investigation integrates GC-MS post-silylation with UHPLC-HRMS/MS and Feature-Based Molecular Networking (FBMN) to achieve deeper coverage of secondary metabolites to more likely account for pepper health benefits. Multivariate statistical analyses were applied to identify key metabolic markers differentiating the two pepper types. Furthermore, the *in vitro* antioxidant and α-glucosidase inhibitory activities were investigated to correlate the bioactivity with the phytochemical profile, offering insights into their functional implications.

## Materials and methods

### Plant material & extraction

Authenticated black and white pepper (*Piper nigrum* L.) entire fruits were kindly provided by Dr Ahmed Mediani, Malaysia from the Institute of Systems Biology, Universiti Kebangsaan Malaysia, Selangor, UKM Bangi, Malaysia, during October 2022. The plant name followed that listed on the plant list website (https://www.theplantlist.org/). The dried fruits were grinded using liquid nitrogen, mortar and pestle and kept at −20 °C till further analyses. A voucher specimen from fruits was deposited at the College of Pharmacy Herbarium (no.: PnB-001/22 and PnW-002/22) Cairo University, Cairo, Egypt. Methanol extract was prepared by cold maceration of pepper samples (10 g each) in 100% MeOH (100 mL) with sonication three times (1 hour for each time) and filtration followed by evaporation under reduced pressure at 45 °C to yield dry residue that was kept at −20 °C until further analyses. For the best assessment of metabolome variations, three independent biological replicates from black and white pepper were analyzed under the same conditions.

### Chemicals

UPLC-MS/MS: Milli-Q water and solvents; formic acid and acetonitrile of LC-MS grade, J. T. Baker (The Netherlands). ABTS: [2,20′-azino-bis(3-ethylbenzothiazoline-6-sulfonic acid)diammonium salt] (≥98% purity). DPPH (2,2-diphenyl-1-picrylhydrazyl), ferric chloride for FRAP (ferric reducing antioxidant power). Trolox (6-hydroxy-2,5,7,8-tetramethyl-chromane-2-carboxylic acid) (≥97% purity). Porcine pancreatic lipase enzyme type 2, intestinal α-glucosidase, orlistat, and acarbose were obtained from Sigma-Aldrich Chemie GmbH, St. Louis, MO.

### GC-MS analysis post silylation

100 μL of the prepared methanol extract^14^ (see plant material) was aliquoted in screw-cap vials and left to evaporate under a nitrogen gas stream until complete dryness. For derivatization, 150 μL of *N*-methyl-*N*-(trimethylsilyl)-trifluoroacetamide (MSTFA) previously diluted at ratio 1 : 1% (v/v) with anhydrous pyridine, was added to the dried methanol extract. The mixture was incubated for 45 min at 60 °C prior to analysis using GC-MS. Separation of silylated derivatives was achieved on a Rtx-5MS (30 m length, 0.25 mm inner diameter and 0.25 m film).^15^ Three biological replicates were extracted and examined in parallel for each specimen under the same conditions. For biological variance assessment, within each specimen and analysis conditions, three independent biological replicates were simultaneously analyzed under same condition. GC-MS analysis was adopted on an Agilent 5977B GC/MSD equipped with a DB-5 column (30 m × 0.25 mm i.d. × 0.25 μm film thickness; Supelco) and coupled to a quadrupole mass spectrometer. The interface and the injector temperatures were both set at 220 °C. Volatile elution was carried out using the following gradient temperature program: oven was set at 40 °C for 3 min, then increased to 180 °C at a rate of 12 °C min^−1^, kept at 180 °C for 5 min, finally increased at a rate of 40 °C min^−1^ to 240 °C and kept at this temperature for 5 min. Helium was utilized as a carrier gas with a total flow rate of 0.9 mL min^−1^.

### GC-MS profiling and modelling of silylated primary metabolites

The protocol to validate silylation was as previously reported.^16^ Soluble sugars, amino acids, organic acids, and fatty acids were quantified using standard curves of glucose, glycine, citric, and stearic acids and the results were expressed as mg g^−1^. Four serial dilutions were prepared from 10 to 600 μg mL^−1^ for establishing the standard curves. Calibration curves for glucose, glycine, citric acid and palmitic acid displayed 0.9948 correlation coefficient (Fig. S1).^17^

Identification of GCMS components was performed by comparing their retention indices (RI) in relation to *n*-alkanes (C6–C20), mass matching to NIST, WILEY library database and with standards if available. Peaks were first deconvoluted using AMDIS software (https://www.amdis.net)^15^ before mass spectral matching.

### UPLC-MS/MS analysis

Finely dried *P. nigrum* fruits (10 mg) were extracted by adding 2 mL of 70% MeOH, containing a 10 μg mL^−1^ umbelliferon as an internal standard and sonicated for 20 min with frequent shaking, then centrifuged at 12 000 × *g* for 10 min to remove debris. The filtered extract through a 0.22 μm filter was subjected to solid-phase extraction using a C_18_ cartridge (Sep Pack, Waters, Milford, MA, USA). Solid-phase extraction was performed to enrich secondary metabolites prior to UPLC-MS/MS analysis. Briefly, 1 mL of methanolic extract was diluted with 4 mL of distilled water and loaded onto pre-conditioned C_18_ SPE cartridges (*e.g.*, Waters Sep-Pak, 500 mg, 6 mL). The cartridges were activated with 5 mL methanol followed by 5 mL water. After sample loading, the cartridges were washed with 5 mL water to remove polar interferences. Elution was performed using 5 mL methanol. The eluates were collected, evaporated under a nitrogen stream, and reconstituted in 200 μL of 80% methanol for UPLC-MS/MS analysis. UPLC-ESI-qTOF-MS analysis was carried out using an ACQUITY UPLC system (Waters, Milford, MA, USA). Chromatographic separation was carried out at 40 °C, using a Waters HSS T3 column (C_18_, 1.0 mm × 100 mm, 1.8 μm) with mobile phases A (0.1% formic acid in water) and B (acetonitrile). The flow rate was set at 0.15 mL min^−1^. The gradient profile was as follows: 0–1 min, 5–5% B; 1–11 min, 5–100% B; 11–19 min, 100% B; 19–20 min, 100–5% B; 20–25 min, 5% B. The analysis was followed as ref. 18. Mass spectrometric detection was carried out on Waters Synapt XS mass spectrometer (Waters Corporation, Milford, USA) equipped with an ESI source. The full scan data were acquired from 50 to 1200 Da, using a capillary voltage of 4.0 kV for positive ion mode and 3.0 kV for negative ion mode, sampling cone voltage of 30 V for positive ion mode and 35 V for negative ion mode, extraction cone voltage of 4.0 V, source temperature of 140 °C, cone gas flow of 50 L h^−1^, desolvation gas (N2) flow of 1000 L h^−1^ and desolvation gas temperature of 450 °C. The collision voltage was set as 5.0 eV for low-energy scan and 25–50 eV for high-energy scan. Data were centroided and mass was corrected during acquisition using an external reference (Lock-Spray™) consisting of a 200 ng mL^−1^ solution of leucine enkephalin infused at a flow rate of 10 μL min^−1^*via* a Lockspray interface, generating a real-time reference ion of [M + H]^+^ (*m*/*z* 556.2771) in positive ion mode and [M − H]^−^ (*m*/*z* 554.2615) in negative ion mode to ensure accurate MS analysis. All data collected in centroid mode were obtained and used to calculate the accurate mass and composition of relative target ions with MassLynx™ V4.2 software (Waters). Three different samples were analysed under the same conditions to evaluate biological replicates. Annotation of metabolites was based on full mass spectra, molecular formula with an (error < 5 ppm), and by comparing fragmentation patterns with available literature, and the phytochemical dictionary of natural products database,^19^ HMDB, FOODB and MASS BANK.

### Molecular networking of UPLC-MS/MS data

Molecular networking (MN) by the GNPS website (https://gnps.ucsd.edu) was performed depending on the UPLC-MS/MS data set for *Piper nigrum* L., black and white samples in both negative and positive ionization modes. The followed parameters were: least cosine score = 0.70; least matched peaks = 4 peaks, parent mass tolerance = 0.02 Da, fragment ion tolerance = 0.02 Da and minimum cluster size was one compound. The MN was visualized by Cytoscape 3.9.1. Compounds spectra were represented in the MN clusters as nodes that connected with each other by edges based on their identified structures and resemblances of their fragmented ions resulting in consensus spectra.^20^ Links of molecular networks are UCSD Computational Mass Spectrometry Website (https://gnps.ucsd.edu/ProteoSAFe/status.jsp?task=395d84b7c1fd48bda89265aa53d45576) and UCSD Computational Mass Spectrometry Website (https://gnps.ucsd.edu/ProteoSAFe/status.jsp?task=395d84b7c1fd48bda89265aa53d45576) for positive and negative modes, respectively.

### Multivariate data analyses of GC-MS and UPLC-MS/MS datasets

Peak abundance from GC-MS and LC-MS was obtained using MS-DIAL software with previously described parameters in ref. 21. Chemometric analysis was done by principal component analysis (PCA) and orthogonal partial least squares discriminate analysis (OPLS-DA) using SIMCA-P software (Version 14.0, Umetrics, Umeå, Sweden). Due to the limited number of biological replicates (*n* = 3 per group), the dataset was not split into independent training and test sets. Model fitness was based on the determination of *R*^2^ (goodness of model fit) and *Q*^2^ (degree of the model predictability). Markers were subsequently identified by analyzing the *S*-plot, which was declared with covariance (*p*) and correlation (*p*_cor_). All variables were mean-centered and scaled to Pareto variance. Model validation was assessed by computing the diagnostic indices, *viz. Q*^2^ and *R*^2^ values, and permutation testing.

### Total phenolics content

Estimation of black and white pepper for total phenolic content (TP) was based on Folin–Ciocâlteu method.^22^ Absorbance was recorded at 760 nm after triplicate measurements. Results were represented as milligrams of gallic acid equivalent per gram of sample (mg GAE per g extract).

### Total flavonoids content

Estimation of total flavonoid content (TF) was done using the aluminium chloride assay.^22^ Absorbance was measured at (415 nm). Results were recorded as milligrams of rutin equivalent per gram the sample (mg RE per g extract), after triple measurements (mean ± SD).

### *In vitro* antioxidant assay

Antioxidant free radical scavenging activity; DPPH (1,1-diphenyl-2-picrylhydrazyl) and ABTS [2,2′-azino-bis(3-ethylbenzothiazoline)6-sulfonic acid] were performed along with ferric reducing capacity (FRAP) following the exact protocol described in ref. 22–24. First, extracts were dissolved in 70% ethanol to get a concentration of 1 mg mL^−1^.

For DPPH free radical scavenging assay, 30 μL of each extract was mixed with a 0.004% methanol solution of DPPH, then incubated for half an hour in the dark at 37 °C.

For ABTS free radical scavenging assay, 2.45 mM potassium persulfate was added to 7 mM ABTS solution to prepare the ABTS^+^ radical solution, incubated in the dark at 37 °C for half a day. Then, the mixture was diluted with distilled water until the absorbance reached 0.70 ± 0.02 at 734 nm then mixed with the extracts and incubated for half an hour at 37 °C.

Finally, for FRAP, the reagent was prepared by mixing 10 : 1 : 1; acetate buffer (0.3 M, pH 3.6), 2,4,6-tris(2-pyridyl)-*s*-triazine (TPTZ) (10 mM) : ferric chloride (20 mM) in 40 mM HCl, then the reagent was mixed with the tested extracts and incubated for half an hour at 37 °C.

A calibration curve was established using different concentrations of a standard antioxidant (Trolox). Results were recorded as mg of Trolox equivalents per gram sample (mg TE per g extract), after triplicates (mean ± SD). Absorbances were recorded in 96-well plates (SPECTROstar® Nano Multi-Detection Microplate Reader; BMG Labtech, Ortenberg, Germany) at 517, 734 & 593 nm for DPPH, ABTS and FRAP assays, respectively.

### *In vitro* pancreatic lipase (PL) type II enzyme inhibition assay

To achieve an enzyme concentration of 200 U mL^−1^ (5 mg mL^−1^), porcine PL type II enzyme was suspended in 2.5 mmol of Tris–HCL buffer at pH 7.4 adjusted by 2.5 mmol NaCl. Samples prepared at a concentrations of 5 and 10 mg mL^−1^ were incubated for (5 min at 37 °C) in 0.1 mL of PL solution, followed by the addition of 10 mM of acetonitrile-dissolved *p*-nitrophenyl butyrate substrate. Inhibition activity was recorded colorimetrically depending on the release of *p*-nitrophenol at 410 nm, the blank consisted of denatured enzyme, prepared according to a modified version of the method described by Bustanji *et al.*.^25^ IC_50_ results were reported in triplicates using orlistat as a standard drug. The equation used for IC_50_ values was: % of enzyme inhibition = (*A*_c_ − *A*_s_/*A*_c_) × 100, where *A*_c_ was control absorbance was *A*_s_ was sample absorbance.

### *In vitro* α-glucosidase enzyme inhibition assay

50 μL of yeast α-glucosidase (1 U mL^−1^) and equal volumes of (A) diluted samples at a concentration of 5 & 10 mg mL^−1^, (B) 100 mM phosphate buffer (pH 6.8) was mixed with (C) the substrate 5 mM *p*-nitrophenol-α-d-glucopyranoside and incubated at 37 °C for 2 min. IC_50_ results were calculated from three readings at 405 nm of color formed after *p*-nitrophenol release. Acarbose was used as standard drug following the exact protocol of.^24,26^ The equation used was % enzyme inhibition = (*A*_c_ − *A*_s_/*A*_c_) × 100, where *A*_c_ was control absorbance and *A*_s_ was sample absorbance.

### Statistical analysis

The data of biological investigations were presented as mean ± standard deviation of three biological replicates. A probability value of *P* < 0.05 was considered to denote the statistically significant differences using the *t*-test.

## Results and discussion

A comparative assessment of primary and secondary metabolites in black and white pepper was performed using a multiplex metabolomic approach *via* GC-MS and UPLC-MS/MS platforms targeting primary and secondary metabolite profiles as explained in the next sections. Chemometric tools were employed to assess similarities as well as differences among black and white pepper.

### GC-MS metabolites profiling of the black and white pepper

GC-MS post-silylation analysis was employed to profile primary metabolites in black and white pepper with a total of 51 peaks (Table 1) belonging to fatty acids/esters (9 peaks), alkaloids/nitrogenous (6 peaks), sugars (3 peaks), sugar alcohols (5 peaks), organic acids (15 peaks), alcohols (4 peaks), and aliphatic hydrocarbons (6 peaks) in addition to phenols (3 peaks).

**Table 1:** Quantitative analysis of silylated primary metabolites (mg g^−1^) in black pepper (BP) and white pepper (WP) *via* GC-MS, *n* = 3^a^

Peak no.	Average *R*_t_ (min)	Average RI	Metabolite	Class	BP (mg g^−1^)	WP (mg g^−1^)
					Mean ± SD	Mean ± SD
1	5.107	1060	Glycolic acid, (2TMS)	Acid	0.24 ± 0.01	0.24 ± 0.01
2	5.202	1066	Lactic acid, (2TMS)	Acid	0.34 ± 0.18	0.35 ± 0.15
3	5.222	1067	Propanoic acid, (TMS)	Acid	0.18 ± 0.01	0.18 ± 0.01
4	5.317	1073	Caproic acid, (TMS)	Acid	0.03 ± 0.00	0.28 ± 0.02
5	5.41	1079	Glycolic acid isomer (2TMS)	Acid	0.03 ± 0.01	0.06 ± 0.00
6	5.712	1097	Octanoic acid, (TMS)	Acid	0.09 ± 0.01	0.10 ± 0.01
7	5.828	1104	Oxalic acid (2TMS)	Acid	2.44 ± 0.50	2.78 ± 0.17
8	6.07	1120	Methylphosphonic acid, (2TMS)	Acid	0.01 ± 0.00	0.01 ± 0.00
9	6.428	1142	β-Lactic acid, (2TMS)	Acid	0.02 ± 0.01	0.03 ± 0.00
14	8.121	1254	Octanoic acid, (TMS)	Acid	0.01 ± 0.01	0.01 ± 0.00
17	8.857	1307	Succinic acid (2TMS)*	Acid	0.05 ± 0.02	0.04 ± 0.01
19	9.19	1331	γ-Aminobutyric acid, (3TMS) isomer	Acid	0.63 ± 0.10	0.06 ± 0.02
23	10.421	1422	Dimethylmalonic acid, (2TMS)	Acid	0.04 ± 0.00	0.05 ± 0.00
24	11.245	1488	Malonic acid (2TMS)	Acid	0.05 ± 0.02	0.04 ± 0.01
**28**	11.791	1532	γ-Aminobutyric acid, (3TMS)	Acid	0.04 ± 0.00	0.05 ± 0.00
Total acids					**4.22** ± **0.87**	**4.26** ± **0.42**
11	6.689	1158	1,4-Butanediol, (2TMS)	Alcohol	0.03 ± 0.00	0.03 ± 0.00
12	7.446	1206	1-Octanol, (TMS)	Alcohol	0.85 ± 0.10	0.61 ± 0.03
39	18.118	2135	1-Octadecanol, (TMS)	Alcohol	0.03 ± 0.00	0.03 ± 0.00
45	21.414	2529	1-Docosanol, (TMS)	Alcohol	0.13 ± 0.04	0.13 ± 0.03
Total alcohol					**1.03** ± **0.14**	**0.80** ± **0.06**
22	9.941	1385	Tetradecane	Aliphatic hydrocarbon	0.08 ± 0.02	0.07 ± 0.00
29	12.413	1582	Hexadecane	Aliphatic hydrocarbon	0.12 ± 0.00	0.12 ± 0.00
31	14.633	1780	Octadecane	Aliphatic hydrocarbon	0.15 ± 0.01	0.15 ± 0.01
35	16.647	1978	Eicosane	Aliphatic hydrocarbon	0.17 ± 0.01	0.16 ± 0.01
40	18.483	2175	Heneicosane	Aliphatic hydrocarbon	0.17 ± 0.01	0.16 ± 0.00
43	20.263	2383	Tetracosane	Aliphatic hydrocarbon	0.02 ± 0.00	0.02 ± 0.00
Total aliphatic hydrocarbon					**0.71** ± **0.05**	**0.68** ± **0.02**
20	9.437	1349	Butyl caprylate	Fatty acid/ester	0.60 ± 0.02	0.61 ± 0.01
25	11.449	1505	Methyl octanoate, (TMS)	Fatty acid/ester	0.12 ± 0.01	0.11 ± 0.00
37	17.111	2026	Palmitic acid, (TMS)	Fatty acid/ester	0.41 ± 0.11	0.38 ± 0.03
41	18.884	2220	Oleic acid, (TMS)	Fatty acid/ester	0.39 ± 0.05	0.41 ± 0.03
44	21.381	2525	α-Hydroxybehenic acid methyl ester, (TMS)	Fatty acid/ester	21.29 ± 21.49	21.06 ± 21.21
**46**	21.754	2572	1-Monopalmitin, (TMS)	Fatty acid/ester	0.40 ± 0.07	0.63 ± 0.37
48	23.181	2767	Monostearin, (2TMS)	Fatty acid/ester	0.16 ± 0.01	0.18 ± 0.04
32	15.171	1831	Myristic acid, (TMS)	Fatty acid/ester	0.08 ± 0.03	0.04 ± 0.01
**42**	19.029	2238	Stearic acid, (TMS)	Fatty acid/ester	0.02 ± 0.00	0.02 ± 0.00
Total fatty acids/ester					**23.48** ± **21.79**	**23.44** ± **21.70**
13	7.755	1228	Ethanolamine, (3TMS)	Nitrogenous	0.06 ± 0.01	0.06 ± 0.00
15	8.247	1263	*N*-(Trimethylsiloxycarbonyl)piperidine	Nitrogenous	0.56 ± 0.03	0.57 ± 0.01
27	11.669	1522	Pipecolinic acid, (2TMS)	Nitrogenous	0.05 ± 0.02	0.08 ± 0.01
Total nitrogenous					**0.67** ± **0.06**	**0.72** ± **0.01**
47	23.044	2748	Piperine isomer*	Nitrogenous (alkaloid)	1.20 ± 0.23	1.80 ± 0.17
**50**	23.96	2850	Piperyline	Nitrogenous (alkaloid)	0.02 ± 0.01	0.06 ± 0.01
51	24.107	2864	Piperine*	Nitrogenous (alkaloid)	3.44 ± 1.45	5.95 ± 0.49
Total nitrogenous (alkaloid)					**4.66** ± **1.69**	**7.81** ± **0.66**
10	6.508	1147	3-Methylphenol, (TMS)	Phenols	0.00 ± 0.00	0.01 ± 0.00
18	8.948	1314	Pyrocatechol, (TMS)	Phenols	0.02 ± 0.00	0.05 ± 0.00
**21**	9.803	1375	Hydroquinone, (2TMS)	Phenols	0.00 ± 0.00	0.03 ± 0.01
Total phenols					**0.02** ± **0.00**	**0.09** ± **0.01**
**16**	8.383	1273	Glycerol, (3TMS) *	Sugar alcohol	0.18 ± 0.02	0.25 ± 0.01
26	11.629	1519	l-Threitol, (4TMS)	Sugar alcohol	0.02 ± 0.01	0.01 ± 0.00
30	14.095	1732	Ribitol, (5TMS)	Sugar alcohol	0.05 ± 0.01	0.02 ± 0.00
34	16.338	1947	Mannitol, (6TMS)-	Sugar alcohol	0.03 ± 0.01	0.00 ± 0.00
**38**	17.854	2107	Myoinositol (TMS)	Sugar alcohol	0.12 ± 0.03	0.02 ± 0.01
Total sugar alcohols					**0.39** ± **0.08**	**0.30** ± **0.03**
33	15.92	1906	d-Glucose, (5TMS) *	Sugar	0.09 ± 0.04	0.04 ± 0.01
36	16.876	2000	d-Glucose, (5TMS) (isomer)	Sugar	0.01 ± 0.01	0.01 ± 0.00
49	23.37	2793	Sucrose, (8TMS) *	Sugar	0.01 ± 0.00	0.03 ± 0.00
Total sugars					**0.11** ± **0.05**	**0.08** ± **0.02**

a Compounds marked with an asterisk (*) were reported in our previous study.^13^

#### Fatty acids or esters

Fatty acids or esters were detected in black and white pepper at high levels of *ca*. 23.4 mg g^−1^. Unsaturated fatty acid represented by oleic acid (peak 41) was detected in both peppers at 0.4 mg g^−1^. Interestingly, a fatty acid ester identified as α-hydroxybenzoic acid methyl ester (peak 44) was detected as the major fatty acid ester in both types at 21.2 mg g^−1^. Fatty acyl esters of hydroxy fatty acids exhibit potential health benefits, including anti-diabetic and anti-inflammatory effects.^27^ Other fatty acyl esters detected at trace levels included 1-monopalmitin (peak 46) and monostearin (peak 48). 1-Monopalmitin is a monoacylglycerol with potential α-glucosidase inhibition activity.^28^

#### Alkaloids or nitrogenous compounds

Alkaloids were detected at comparably high levels in both pepper samples, with white pepper containing 7.8 mg g^−1^ and black pepper 4.6 mg g^−1^. Piperine, the principal alkaloid in both black and white pepper, was quantified at higher concentrations in white pepper (5.9 mg g^−1^) than in black pepper (3.4 mg g^−1^). These findings were further supported by piperine quantification using quantitative ^1^H NMR spectroscopy.^13^ The piperine level obtained *via* GC-MS post-silylation showed same pattern as observed using NMR spectroscopy being more enriched in white pepper, though with different absolute levels. The variation in piperine quantification may be attributed to differences in analytical platforms, derivatization efficiency, and ionization behavior, especially that piperine content was not based on exact standard but relative to nitrogenous compounds. As NMR spectroscopy is a universal detection method, it is often considered more accurate and reliable than GC-MS for absolute quantification of metabolites. Several biological activities were reported for piperine, including antitumor, antiangiogenesis, antioxidant, and antidiabetic.^29^ Other detected nitrogenous compounds included ethanolamine (peak 13) and pipecolinic acid (peak 27) though at trace levels.

#### Organic acids & alcohols

Organic acids that contribute to the taste and preservative effect were detected in black and white pepper at *ca.* 4.2 mg g^−1^. The organic acid content in fruits play a key role in its dietary importance.^30^ Oxalic acid (peak 7) was detected as the major form at 2.4–2.8 mg g^−1^. Oxalic acid exerts potential antioxidant capacity and can improve fruit quality, enhance crop yield, boost nutritional profile, and delay postharvest senescence in fruit.^31^ Other less abundant organic acids included glycolic acid (peak 1), lactic acid (peak 2), and malonic acid (peak 24). Compared with acids, alcohols were detected at relatively low levels of 0.8–1.03 mg g^−1^ represented mainly by 1-octanol (peak 12) and 1-docosanol (peak 45).

#### Sugars & sugar alcohols

Sugars were detected at trace levels in both peppers at 0.1 mg g^−1^ and represented by glucose (peaks 33 and 36) and sucrose (peak 49). Likewise, sugar alcohols were detected at low levels at 0.3–0.4 mg g^−1^, represented by 5 peaks, including glycerol (peak 16) and myoinositol (peak 38). Sugar alcohols play a key role in the nutritional value and improving taste, being considered as sweeteners without the induction of blood glucose levels.^32^

#### Aliphatic hydrocarbons and phenols

Aliphatic hydrocarbons represented by 6 peaks were detected in both peppers at *ca.* 0.7 mg g^−1^. Likewise, phenolics were detected at trace levels represented by 3-methylphenol (peak 10), pyrocatechol (peak 18), and hydroquinone (peak 21). Profiling of high molecular weight phenolics is more suited to using LC/MS as explained in the next sections.

### Multivariate PCA and OPLS-DA analyses of black and white pepper dataset *via* GC-MS post-silylation

**Fig. 1:**
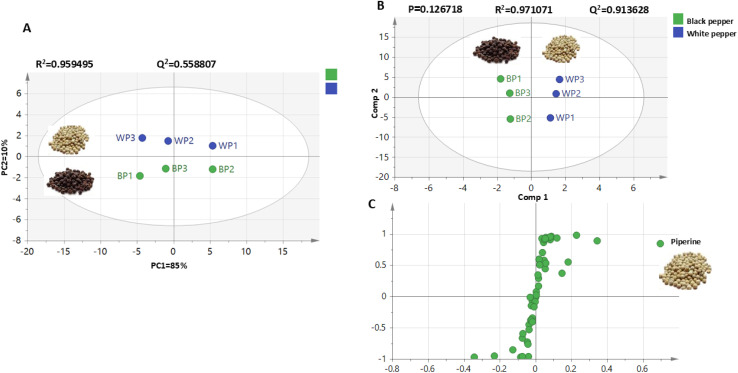
Unsupervised and supervised multivariate data analysis for black and white pepper samples analyzed using GC-MS post-silylation. (A) PCA score plot. (B) OPLS-DA score plot of black and white pepper samples. (C) *S*-Plot derived from modeling black and white pepper silylated metabolites using GC-MS showing the covariance *p* (1) against the correlation *p*(cor) (1) of the variables of the discriminating component of the OPLS-DA model.

Multivariate data analysis was employed to assess the distribution of metabolites among black and white pepper extracts based on GC-MS post-silylation profiling (Fig. 1). A PCA model (Fig. 1A) revealed a clear separation between the two types of pepper, where white pepper clustered at the positive upper side of PC2, *versus* black pepper at the negative side of the PC2 score plot. To further enhance group discrimination, supervised orthogonal projection to latent structures discriminant analysis (OPLS-DA) was further employed to classify between black and white pepper (Fig. 1B). OPLS-DA showed a clear segregation, with black pepper samples positioned to the left side, while white pepper was segregated toward the right side of score plot. The corresponding *S*-plot (Fig. 1C) highlighted piperine (peak 51) as a key discriminant metabolite was enriched in white pepper fruits. OPLS-DA model exhibited strong statistical performance, with a total variance coverage of 97% (*R*^2^ = 0.97) with high prediction power as manifested by *Q*^2^ = 0.91, and suggestive of the higher alkaloid content in white pepper. However, the relatively small number of biological replicates (*n* = 3 per group) may limit the statistical power and generalizability of the multivariate models. While OPLS-DA provided valuable exploratory insights, the model's predictive performance was assessed *via* internal 7 K cross-validation rather than independent test set validation.

### Univariate statistical analysis

GC-MS semi-quantitative results were further statistically analyzed using *t*-test or marker metabolites in black and white pepper (Table S1), with results presented as mean ± standard deviation of the mean (SD). *t*-Test was used to test significance of data and values with *p* < 0.05 are well-thought-out significantly different. Marker metabolites identified to be significant are γ-aminobutyric acid, (3TMS) isomer and myoinositol (TMS).

### UPLC-MS/MS based metabolites profiling of black and white pepper fruit

**Fig. 2:**
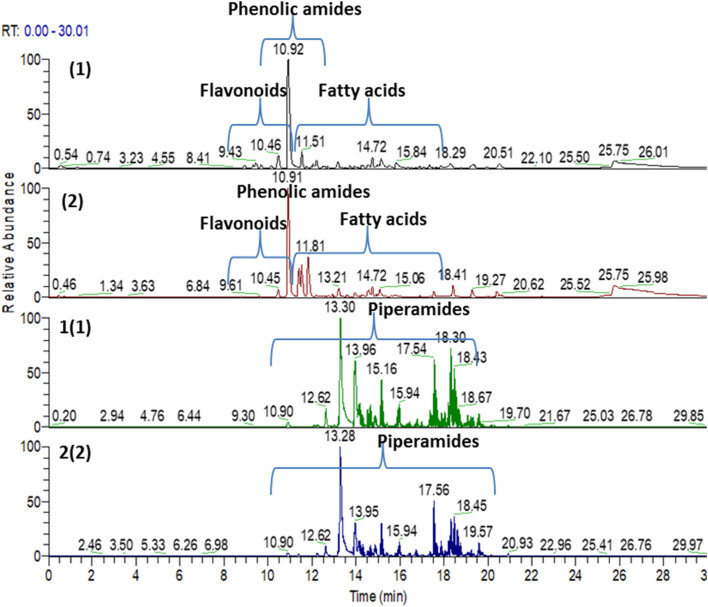
Base peak UPLC-MS/MS chromatograms of black and white *pepper nigrum* L.; (1) Black pepper negative mode, (2) white pepper negative mode, 1 (1) black pepper positive mode, 2 (2) white pepper positive mode.

**Fig. 3:**
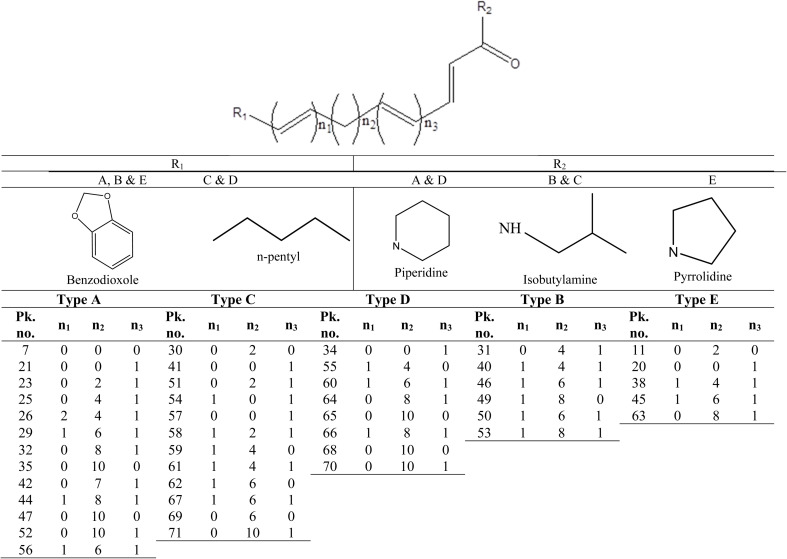
Structures of identified piperamides different types (A–E), characterizing the two tails of each type (R1 & R2) and demonstrating compounds' peak numbers detected for each type in the two pepper samples.

**Fig. 4:**
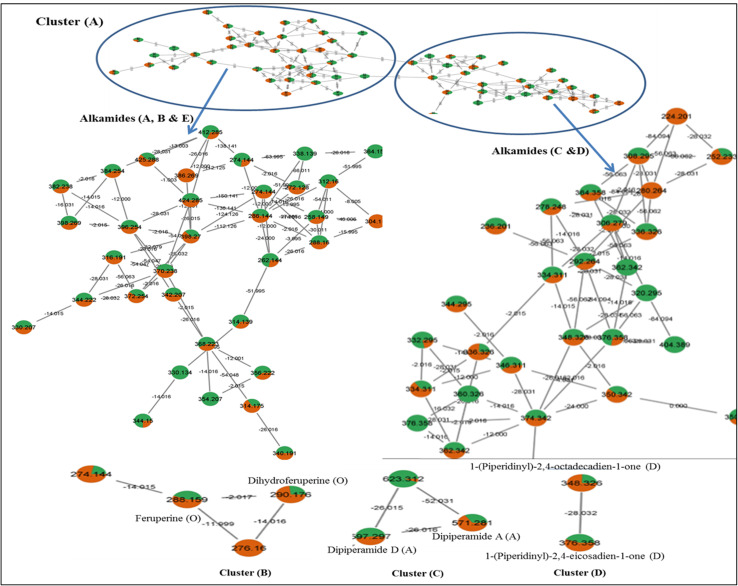
GNPS positive ionization mode clusters of alkaloids detected. Pie charts nodes demonstrated black pepper sample in green color and white pepper in orange color that display the proportion of each metabolite in the two samples.

**Fig. 5:**
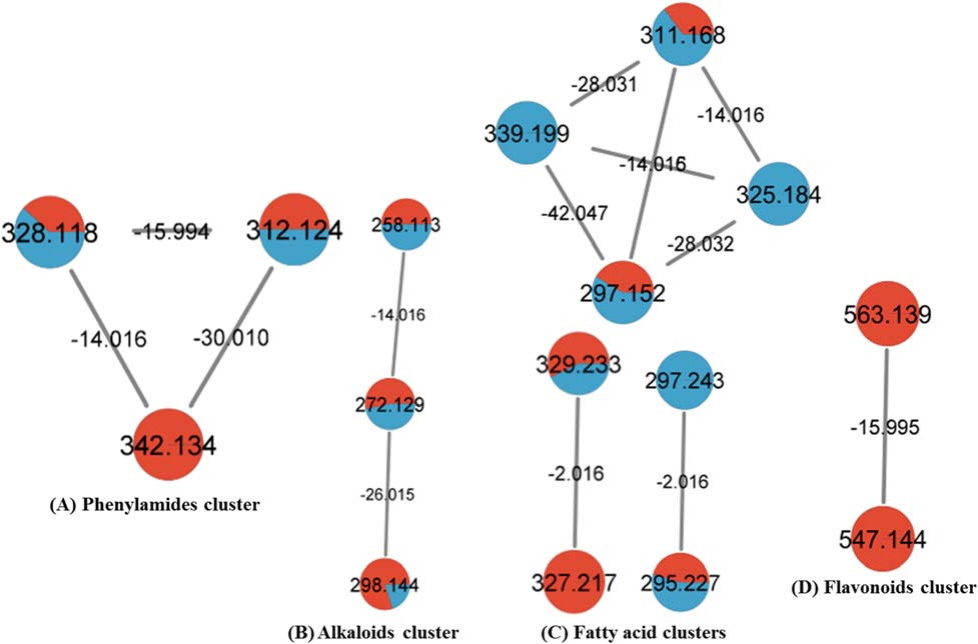
GNPS negative ionization mode clusters of some phenylamides (A), alkaloids (B), fatty acids (C) and flavonoids (D). Pie charts nodes were assigned with red and blue colors for black and white pepper samples, respectively that demonstrated the proportion of each metabolite in the two samples.

UPLC-ESI-qTOF-MS/MS analysis was carried out for black and white pepper profiling targeting more secondary metabolites using dual electrospray ionization modes (positive & negative) (Fig. 2), in contrary to previous studies concerning one mode only of *Piper nigrum* MS/MS analysis.^33,34^ Compounds were eluted within 20 min from the most polar to the least polar according to the reversed phase sequence of elution.^35^ The identification was depended on comparison of the high-resolution mass spectra information with phytochemical dictionary of natural product database and MS/MS online free mass data bases; HMDB, FOODB, MASS BANK and by comparison with the published literature. Spectral data of annotated metabolites were represented in Table 2 showing their relative retention times, molecular ions, molecular formulas and fragment ions. Structures of piperamides, the major class, were illustrated in Fig. 3 that demonstrated number of compounds in each type and distribution of the different types of piperamides over peaks numbers. For assessing the HR-MS/MS dataset, a molecular networking (GNPS) was employed and visualized using “Cytoscape”. Metabolites distribution in the molecular networking aided confirmation and visualization of annotated peaks.^36^ Two molecular networks (MNs) were derived for white and black pepper LC/MS data relative to the dual ionization modes *viz.* negative and positive. Pie charts nodes demonstrated in the network display the proportion of each metabolite in the two samples, nodes were colored for coding the samples; black pepper sample was assigned with green & red colors in positive and negative NWs respectively, while white pepper sample was assigned by orange & blue colors in positive and negative NWs respectively. In positive NW there were 104 nodes & 172 edges with representing most alkaloid fragments clusters, some were illustrated in Fig. 4 (clusters A, B, C & D) while for negative ionization mode NW, 89 nodes & 91 edges were revealed belonging for cluster mostly composed of phenylamides, fatty acids, flavonoids and some alkaloids (Fig. 5 clusters A, B, C & D), and highlighting the benefit of acquiring in both ion modes.

**Table 2:** Identified secondary metabolites in black and white pepper fruits as analyzed *via* UPLC-MS/MS in negative and positive modes^a^

Peak no.	*R*_t_	Mol. ion *m*/*z* (−/+)	Formula	Fragment	Error (ppm)	Compound name	Class	BP	WP	Ref.
1	9.4	595.1668	C_27_H_31_O_15_^+^	449, 287	1.8	Kampferol-*O*-rhamnosyl hexoside	Flavonoid *O*-glycoside	+	—	
2	9.6	577.1547	C_27_H_29_O_14_^−^	457, 431, 311, 413, 293, 323, 269	−0.8	Vitexin-*O*-rhamnoside	Flavonoid *C*–*O*-diglycoside	+	—	
3	9.61	344.1129	C_18_H_18_NO_6_^−^	326, 313, 311	0.5	Di-hydroxy feruloyltyramine	Phenolic amides	+	+	
4	10.13	563.1389	C_26_H_27_O_14_^−^	417, 285	−0.9	Kampferol-*O*-rhamnosyl pentoside	Flavonoid *O*-glycoside	+	—	
5	10.53	547.1442	C_26_H_27_O_13_^−^	401, 311, 269	−0.5	Apigenin-*O*-rhamnosyl pentoside	Flavonoid *O*-glycoside	+	—	
6	10.57	328.1183	C_18_H_18_NO_5_^−^	313, 175, 161, 135	1.1	Hydroxy feruloyltyramine	Phenolic amides	+	+	
7	10.82	258.1130	C_15_H_16_NO_3_^−^	216, 187, 161, 159, 146, 145, 143	2.2	1-[3-(1,3-Benzodioxol-5-yl)-1-oxo-2-propenyl] piperidine	Type A piperamide	+	+	38
8	10.86	312.1234	C_18_H_18_NO_4_^−^	297, 270, 253, 190, 178, 148, 135	1.2	Feruloyltyramine	Phenolic amides	+	+	46
9	11.04	344.1497	C_19_H_22_NO_5_^+^	326, 314, 207, 177, 145, 131, 117	1.5	Sinapoyltyramine	Phenolic amides derv	+	+	51
10	11.07	342.1340	C_19_H_20_NO_5_^−^	327, 328, 312	1.1	Methoxy feruloyltyramine	Phenolic amides	+	—	
11	11.48	274.1445	C_16_H_20_NO_3_^+^	203, 189, 171, 161, 152, 147, 123, 112	−1.1	5-(1,3-Benzodioxol-5-yl)-1-pyrrolidin-1-ylpenten-1-one	Type E piperamide	+	+	
12	11.78	297.1127	C_18_H_17_O_4_^−^	253, 251, 225, 189, 165, 145, 121, 107	1.9	3,4-Bis[(4-hydroxyphenyl) methyl]oxolan-2-one	Lignan	—	+	
13	11.83	327.2170	C_18_H_31_O_5_^−^	309, 291, 269, 251, 229, 211, 183, 171	1.2	Trihydroxy-octadecadienoic acid	Fatty acid	+	—	
14	11.99	331.2483	C_18_H_35_O_5_^−^	313, 295, 255, 171	1.2	Trihydroxy-octadecanoic acid	Fatty acid	+	—	
15	12.02	290.1756	C_17_H_24_NO_3_^+^	272, 258, 205, 177, 137, 154, 163	2.1	Dihydroferuperine	Type O piperamide	+	+	
16	12.04	329.2326	C_18_H_33_O_5_^−^	311, 293, 229, 211, 171	1.1	Trihydroxy-octadecenoic acid	Fatty acid	+	+	
17	12.17	258.1493	C_16_H_20_NO_2_^+^	173	1.3	Coumaperine	Type O piperamide	+	+	62
18	12.18	288.1600	C_17_H_22_NO_3_^+^	203	−2.1	Feruperine	Type O piperamide	+	+	34
19	12.19	298.1443	C_18_H_20_NO_3_^−^	253, 213, 185, 159, 146, 121, 109	2	Acetyl coumaperine	Type O piperamide	+	+	
20	12.55	272.1290	C_16_H_18_NO_3_^+^	201, 173, 143, 135, 115	3.4	Piperyline	Type E piperamide	+	+	33
21	13.2	288.1595	C_17_H_22_NO_3_^+^	203, 175, 161, 135, 112	0.34	Piperanine	Type A piperamide	—	+	33
22	13.34	313.2377	C_18_H_33_O_4_^−^	297, 277, 267, 239, 171	1.4	Dihydroxy-12-octadecenoic acid	Fatty acid	+	+	
23	13.4	286.1440	C_17_H_20_NO_3_^+^	201, 135, 173, 161	0.9	Piperine*	Type A piperamide	+	+	34
24	13.81	280.1914	C_16_H_26_NO_3_^−^	262, 236, 194, 177	4.5	Unknown	—	+	—	
25	13.89	314.1754	C_19_H_24_NO_3_^+^	229, 201, 161, 135, 112	1.1	Piperdardine/pipersintenamide	Type A piperamide	—	+	38
26	13.95	312.1598	C_19_H_22_NO_3_^+^	227, 199, 164, 161, 112	1.4	Piperettine	Type A piperamide	—	+	33
27	14.02	297.1524	C_12_H_25_O_8_^−^	325, 279, 233, 212, 198, 183	−1.9	Unknown	Fatty acid oligomer	+	—	
28	14.22	293.2117	C_18_H_29_O_3_^−^	275, 265, 235, 211, 171	0.6	Hydroxy-octadecatrienoic acid	Fatty acid	+	+	
29	14.26	316.1910	C_19_H_26_NO_3_^+^	231, 213, 201, 294, 135	1.1	Piperolein A	Type A piperamide	+	+	63
30	14.27	224.2014	C_14_H_26_NO^+^	151, 133, 109, 95	2.6	Pellitorine	Type C piperamide	—	+	33 and 34
31	14.48	330.2067	C_20_H_28_NO_3_^+^	257, 229, 161, 135	1.2	Pipercallosine	Type B piperamide	+	+	33 and 34
32	14.6	340.1909	C_21_H_26_NO_3_^+^	255, 227, 179, 218, 161, 131	0.6	Dehydropipernonaline	Type A piperamide	+	+	33 and 34
33	14.71	295.2272	C_18_H_31_O_3_^−^	277, 263, 251, 195, 183, 171	0.4	Hydroxy-octadecadienoic acid	Fatty acid	+	+	
34	14.81	236.2003	C_15_H_26_NO^+^	208, 151, 133, 109, 95	−2.1	Neopellitorine B	Type D piperamide	+	+	33
35	14.82	342.2064	C_21_H_28_NO_3_^+^	257, 229, 199, 161	0.3	Pipernonaline	Type A piperamide	+	+	33
36	14.9	297.2429	C_18_H_33_O_3_^−^	279, 251, 223, 171	1.4	Hydroxy-octadecenoic acid	Fatty acid	—	+	
37	14.9	571.2807	C_34_H_39_N_2_O_6_^+^	486, 458	1.6	Dipiperamide A	Type A piperamide	+	+	37
38	14.95	354.2066	C_22_H_28_NO_3_^+^	283, 265, 255, 161, 95	0.8	1-(Pyrrolidinyl)-11-(3′,4′-methylenedioxyphenyl)-2,4,10-undecatrien-1-one	Type E piperamide	+	—	33
39	15.01	597.2965	C_36_H_41_N_2_O_6_^+^	512, 484, 312, 286	1.4	Dipiperamide D	Type A piperamide	+	+	37
40	15.09	356.2227	C_22_H_30_NO_3_^+^	283, 255, 215, 161, 135	2	Retrofractamide B	Type B piperamide	—	+	33 and 34
41	15.34	252.2324	C_16_H_30_NO^+^	119, 121, 133, 135, 196, 179, 161, 151, 95	1.1	*N*-Isobutyl-2,4-dodecadienamide	Type C piperamides	+	+	33 and 34
42	15.51	368.2226	C_23_H_30_NO_3_^+^	283, 255, 227, 215, 161	1.5	Piperundecalidine	Type A	+	+	33
43	15.72	339.1991	C_15_H_31_O_8_^−^	321, 295, 275, 267, 253, 239, 225, 211, 197, 183, 170	−6	Hexahydroxy-pentadecanoic acid	Fatty acid oligomer	—	+	
44	15.75	370.2382	C_23_H_32_NO_3_^+^	285, 267, 257, 175, 161, 135	−4	Piperchabamide B	Type A piperamide	+	+	33 and 34
45	15.84	382.2381	C_24_H_32_NO_3_^+^	313, 283, 135	1.2	Brachyamide A	Type E piperamide	+	+	33
46	16.10	384.2537	C_24_H_34_NO_3_^+^	311, 283, 161	1.1	Guineensine	Type B piperamide	+	+	33 and 34
47	16.12	372.2538	C_23_H_34_NO_3_^+^	287, 269, 250, 177, 161, 135	1.3	11-(Benzo[1,3]dioxol-5-yl)-1-(piperidin-1-yl) undecaen-1-one	Type A piperamide	+	+	
48	16.16	623.3119	C_25_H_51_O_17_^+^	538, 471, 453, 425, 336, 312	−0.2	Unknown	—	+	+	
49	16.20	386.2688	C_24_H_36_NO_3_^+^	313, 285, 161, 135	−0.2	Piperflaviflorine	Type B piperamide	—	+	33
50	16.29	398.2689	C_25_H_36_NO_3_^+^	311, 283, 269, 161, 135	−0.21	Pipwaqarine	Type B piperamide	+	+	39
51	16.39	280.2640	C_18_H_34_NO^+^	119, 121, 135, 135, 224, 207, 189, 182, 179	1.9	Pipilyasine	Type C piperamide	—	+	34 and 64
52	16.40	396.2542	C_25_H_34_NO_3_^+^	383, 311, 161	2.3	Piperchabamide C	Type A piperamide	+	+	33 and 34
53	16.76	412.2847	C_26_H_38_NO_3_^+^	339, 311, 161	0.3	Brachystamide B	Type B piperamide	+	+	33 and 34
54	16.92	332.2954	C_22_H_38_NO^+^	119, 121, 133, 135, 259, 231, 161, 135	0.6	*N*-Isobutyl-2,4,10,12-octadecatetraenamide	Type C piperamide	+	+	33
55	16.96	292.2633	C_19_H_34_NO^+^	119, 121, 133, 135, 207, 189	−0.5	1-(Piperidinyl)-2,8-tetradecadien-1-one	Type D piperamide	+	+	33 and 34
56	17.22	344.2226	C_21_H_30_NO_3_^+^	259, 231, 161, 135	7.5	Unidentified	Type A piperamide	+	—	33 and 34
57	17.67	334.3101	C_22_H_40_NO^+^	119, 121, 133, 135, 261, 233, 135, 109	−0.9	*N*-Isobutyl-2,4,12-octadecatrienamide	Type C piperamide	+	+	33 and 34
58	17.8	336.3262	C_22_H_42_NO^+^	318, 263, 280, 294, 266, 261, 145, 224, 221, 135	0.5	*N*-Isobutyl-2,4-octadecadienamide	Type C piperamide	+	+	33 and 34
59	18.1	338.3423	C_22_H_44_NO^+^	121, 135, 296, 282, 265, 221, 247, 212, 135	1.7	*N*-Isobutyl-octadecenamide	Type C piperamide	+	+	65
60	18.2	346.3103	C_23_H_40_NO^+^	119, 121, 133, 135, 318, 290, 261, 233, 350, 243, 135, 112	−0.6	1-(Piperidinyl)-2,4,12-octadecatrien-1-one	Type D piperamide	+	+	33 and 34
61	18.3	336.3264	C_22_H_42_NO^+^	119, 121, 133, 135, 263, 280, 235	0.9	Pipzubedine	Type C piperamide	+	+	64
62	18.49	362.3421	C_24_H_44_NO^+^	119, 121, 133, 135, 308, 289, 261, 271, 320, 306, 251, 233, 135, 109	0.9	*N*-Isobutyl-2,4,14-eicosatrienamide	Type C piperamide	+	+	33 and 34
63	18.5	334.3106	C_22_H_40_NO^+^	263, 231, 316, 161, 152, 135, 119	0.6	Pipyaqubine	Type E piperamide	+	—	64
64	18.59	348.3265	C_23_H_42_NO^+^	135, 263, 245, 235, 133, 112	1.3	1-(Piperidinyl)-2,4-octadecadien-1-one	Type D piperamide	+	+	33 and 34
65	19.04	350.3422	C_23_H_44_NO^+^	294, 308, 112, 121, 135, 266, 252, 238, 224, 121, 182, 186, 154	1.3	1-(Piperidinyl)-octadecen-1-one	Type D piperamide	+	+	66
66	19.18	374.3425	C_25_H_44_NO^+^	121, 135, 289, 318, 304, 271, 261	−0.27	1-(Piperidinyl)-2,4,14-eicosatrien-1-one	Type D piperamide	+	+	33 and 34
67	19.30	364.3569	C_24_H_46_NO^+^	119, 121, 308, 291, 273	−1.3	2,4-Eicosadienoic acid methylpropylamide (*N*-Isobutyl-2,4-eicosadienamide)	Type C piperamide	+	+	33 and 34
68	19.53	350.3414	C_23_H_44_NO^+^	112, 121, 135, 350, 265, 182	1.3	Pipercitine	Type D piperamide	+	+	66
69	19.67	362.3416	C_24_H_44_NO^+^	121, 135, 308, 273, 291, 289, 262	−0.28	*N*-(4-Methylpentyl)-2,4,12-octadecatrienamide	Type C piperamide	+	—	67
70	20.26	376.3576	C_25_H_46_NO^+^	119, 133, 348, 322, 291, 273	1.8	1-(Piperidinyl)-2,4-eicosadien-1-one	Type D piperamide	+	+	33 and 34
71	20.8	378.3729	C_25_H_48_NO^+^	112, 322, 294, 252, 210, 182, 154	−0.3	Pipkirine	Type C piperamide			68

a Compounds marked with an asterisk (*) were reported in our previous study.^13^

A total of 71 metabolites were tentatively identified belonging to different classes: piperamides (the most abundant class), flavonoids, hydroxycinnamic acids, fatty acids and a lignin some were detected for the first time from the plant as mentioned with explanation for each class is in the next subsections.

#### Alkaloids (piperamides)

The most abundant class in examined black and white *P. nigrum* L. samples was piperamides that is readily detected in UPLC-MS/MS positive ionization mode. Piperamides are classified into 6 different types; A, B, C, D, E & O, and all identified compounds are listed in Table 2. The structure of piperamide is constituted mainly from two main groups characterizing the type of piperamide that is attached as two tails on a chain of fatty acid which differs in number of carbon atoms and double bonds. One of the two tails is nitrogenous that condenses with the fatty acyl moiety forming an amide linkage. In type A and D the nitrogenous end is piperidine, whereas, in types B and C this end is isobutylamine, while, for type E, it is a pyrrolidine unit. The other tail attached to the piperamide structure is benzodioxole as in types A, B and E (aromatic nucleus) or *n*-pentyl group as in types C and D. Structures of piperamide alkaloids were illustrated in Fig. 3 demonstrating the numbers of carbon atoms and double bonds in the fatty acid chain of alkamides of different types with recording compounds peaks numbers for each type. Other different structures are collected in type O as previously called.^33^ Different types of piperamides were detected in positive MN (Fig. 4A–D) and negative MN (Fig. 5B).

Among piperamides, type A was the most abundant type, 13 piperamides of type A (peaks 7, 21, 23, 25, 26, 29, 32, 35, 42, 44, 47, 52 & 56) were detected in both pepper samples (Fig. S2) except for piperdardine (C_19_H_24_NO_3_^+^) at *m*/*z* 314.1754 was found in white pepper, only in contrary to unidentified alkaloid (C_21_H_30_NO_3_^+^) at *m*/*z* 344.2226 that was detected exclusively in black pepper sample. Two bisalkaloids (peaks 37& 39); dipiperamides A (C_34_H_39_N_2_O_6_^+^) & D (C_36_H_41_N_2_O_6_^+^) were annotated at *m*/*z* 571.2807 & 597.2965, respectively from our two pepper samples, the former was isolated previously from white pepper with another two compounds; dipiperamides B & C.^37^ 1-[3-(1,3-Benzodioxol-5-yl)-1-oxo-2-propenyl]piperidine (peak 7, C_15_H_16_NO_3_^−^) was reported before from root^38^ but first to be detected in *Piper nigrum* fruit appeared at *m*/*z* 258.1130, while another alkaloid of type A; 11-(benzo[1,3]dioxol-5-yl)-1-(piperidin-1-yl)undecaen-1-one (peak 47, C_23_H_34_NO_3_^+^) was first time to be identified as our knowledge from *Piper nigrum* appeared at *m*/*z* 372.2538. Piperamides of type A were demonstrated in positive MN clusters A & C in Fig. 4.

The second abundant type of piperamides was type C represented by 12 alkaloids (peaks 30, 41, 51, 54, 57–59, 61, 62, 67, 69 & 71) mostly from both pepper samples except *N*-isobutyl-2,4,14-eicosatrienamide (C_24_H_44_NO^+^) detected at *m*/*z* 362.3421 (Fig. S3), pellitorine (C_14_H_26_NO^+^) at *m*/*z* 224.2014 and pipilyasine (C_18_H_34_NO^+^) at *m*/*z* 280.2640 found mostly, in white pepper sample, and in contrast to *N*-(4-methylpentyl)-octadecatrienamide (C_24_H_44_NO^+^) at *m*/*z* 362.3416 that was not detected in the white pepper, some alkamides of the C were demonstrated in positive MN as shown in Fig. 4 cluster A.

For type D, 8 alkaloids (peaks 34, 55, 60, 64–66, 68 & 70) were annotated in the two peppers (Fig. S4) most of them were previously reported from *P. nigrum*,^33^ some alkaloids of type D were represented in positive MN (Fig. 4A and D).

Six piperamides of type B (peaks 31, 40, 46, 49, 50, 53) were identified from both white and black pepper samples except for retrofractamide B (C_22_H_30_NO_3_^+^) & piperflaviflorine (C_24_H_36_NO_3_^+^) at *m*/*z* 356.2227 and 386.2688 (Fig. S5), respectively that were not detected in white pepper. Piperamides of type B are reported before from *P. nigrum* and allied species.^33,39^ Type B was demonstrated herein in positive MN (Fig. 4A).

Regarding type E, 5 piperamides (peaks 11, 20, 38, 45, 63) were detected in both samples except for 1-(pyrrolidinyl)-11-(3′,4′-methylenedioxyphenyl)-2,4,10-undecatrien-1-one (C_22_H_28_NO_3_^+^) at *m*/*z* 354.2066 that was found in white pepper only with 1-(1,3-benzodioxol-5-yl)-3-pyrrolidin-1-ylpenten-1-one (peak 11, C_16_H_20_NO_3_^+^) first to be detected, to the best of our knowledge, in *P. nigrum* appeared at *m*/*z* 274.1445, see (Fig. 4A and 5B). Fragmentation pattern of Fig. S5 of brachyamide A (type E piperamide) was represented in Fig. S6.

Other pepper alkamides than these mentioned types was O-type, with 4 other alkaloids (peaks 15 & 17–19) identified in our study all of the same parent alkaloid coumaperine (C_16_H_20_NO_2_^+^) (Fig. S7) and two derivatives with mass differences of 30 amu for methoxy substitution (feruperine, C_17_H_22_NO_3_^+^) were detected in positive ionization mode at *m*/*z* 288.1600 and 40 amu for its acetyl derivative detected in the different mode at *m*/*z* 298.1443 (C_18_H_20_NO_3_^−^). These two coumaperine derivatives are first to be detected in *P. nigrum*, along with dihydro feruperine at *m*/*z* 290.1756 (C_17_H_24_NO_3_^+^). All type O were detected in both samples and this type were clustered in the two modes (positive and negative) MNs (Fig. 4B and 5B), respectively.

Different types of piperamides were annotated based on their characteristic fragmentation pattern, as in types A & D amide bond cleavage of the amine part with decarboxylation resulting in neutral loss of piperidine (C_5_H_11_N) and formylpiperdine (C_6_H_11_ON) moieties that showed product ions of [M + H − 85]^+^ & [M + H − 113]^+^ respectively. Regarding the fragmentation pattern of types B & C, neutral loss of isobutylamide (C_4_H_11_N) and formylisobutylamide (C_5_H_11_ON) leads to fragment ions of [M + H − 73]^+^ & [M + H − 101]^+^ respectively. Moreover, in type E the neutral loss of pyrrolidine (C_4_H_9_N) and formylpyrrolidine (C_5_H_9_ON) units showed products ions resulting from [M + H − 71]^+^ & [M + H − 99]^+^ respectively. Furthermore, the other end group loss in types (A, B & E), resulting in product ions at *m*/*z* 161 &135, respective to methylbenzodioxole [C_10_H_9_O_2_ + H]^+^ and propenylbenzodioxole [C_8_H_7_O_2_ + H]^+^ ions after benzodioxole group cleavage and confirming alkaloid structure type. For further information Fig. 3 illustrates the structures of different piperamides types identified herein along with the peak numbers in each type.

Piperamides as major principal components found in *P. nigrum* may affect pepper flavour through synergistic interaction. Piperine (peak 23), the primary bioactive alkaloid, is responsible for the pungent taste and spicy flavor that prompts heat sensation when contact sensory neurons receptors.^40^ Besides, dimeric amides (peaks 37 & 39) may enhance the pungency taste of pepper.^41^ As a conclusion, *P. nigrum* could be used in culinary applications because of bitter, heat and pungent taste sensations that occur by the interaction of many alkaloids, besides their antioxidant and anti-inflammatory effects that add value to pepper as flavor enhancer and nutraceutical.^41,42^

#### Flavonoids

Four *O* & *C*-flavonoid glycosides were identified showing different fragmentation pattern that distinguished between the two types of glycosidic linkages. Peaks 1, 4 and 5 were assigned for two flavonols and one flavone *O*-glycosides, respectively with neutral loss of sugar moieties; [M + H]-[162, 146 & 132 amu] assigned for precursor ions loss of (hexose, deoxy hexose & pentose units), respectively. Peaks 1, 4 & 5 were identified as kaempferol-*O*-rhamnosyl hexoside, kampferol-*O*-rhamnosyl pentoside (Fig. S8) & apigenin-*O*-rhamnosyl pentoside, respectively (Fig. 5, cluster D). While peak 2 appeared at *m*/*z* [M − H]^−^ 577.1547 (C_27_H_29_O_14_)-demonstrating another fragmentation pattern characteristic of *C*-glycosidic linkage showing [M − H − 18 amu] resulting from loss of water molecule and fragments of [M − H − 120 & 90 amu] due to (0.2 and 0.3 cross ring cleavages of hexose unit),^43^ and annotated as vitexin-*O*-rhamnoside, apigenin and kaempferol aglycons were reported before,^44^ but it is the first time, as well as we know, to identify these glycosidic linkages in this species.

#### Hydroxycinnamic acid amides (phenolamides derivatives)

Five peaks (3, 6, 8, 9 &10) were identified as hydroxycinnamic acid amides or phenolamides derivatives, a class that is well known in planta and less common in edible plants.^45^ Peak 8 was annotated as *N*-feruloyltyramine with molecular ion [M − H]^−^ at *m*/*z* 312.1234 (C_18_H_18_NO_4_^−^) (Fig. S9) and characteristic ion fragments as reported by ref. 46. *N*-Feruloyl tyramine was previously reported from white pepper fruits along with another phenolic amide *N*-5-(4-hydroxyphenyl)-2, 4-pentadienoyl piperidine^47^ and from black pepper.^48^ Moreover, peaks 3, 6 & 10 are closely related to *N*-feruloyltyramine with additive mass weight of 32, 16 & 30 amu relative to dihydroxy, hydroxy and methoxy groups first time to be detected from pepper and demonstrated in MN of negative mode (Fig. 5, cluster A). All phenylamides were identified in both pepper samples except methoxy-feruloyltyramine (peak 10) which was detected in black pepper only. Phenylamides account for *P. nigrum* health benefits including its antioxidant effect in neurodegeneration disorders and anticancer effects.^49,50^ Another hydroxycinnamic acyl amide (peak 9) is first to be reported in pepper detected in positive ion mode [M + H]^+^ at *m*/*z* 344.1497 (C_19_H_22_NO_5_^+^) annotated as sinapoyltyramine with a characteristic base peak at *m*/*z* = 145.^51^

#### Fatty acids

The second most abundant class in pepper included fatty acids mostly detected in negative ionization mode due to freely ionized carboxylate groups.^35^ Closely related fatty acids were clustered in negative MN based on MS/MS data (Fig. 5C). Fatty acids with C-18 were annotated showing different saturation and hydroxylation positions; poly-hydroxylated fatty acids (peaks 13, 14 and 16) were identified at *m*/*z* 327.2170, 331.2483 and 329.2326 (C_18_H_31_O_5_^−^, C_18_H_35_O_5_^−^ & C_18_H_33_O_5_^−^) respectively, corresponding to trihydroxy-octadecadienoic acid (Fig. S10), trihydroxy-octadecanoic acid & trihydroxy-octadecenoic, with a mass difference of 2 amu relative to extra double bonds. Additionally, mono-hydroxylated fatty acids also demonstrated different degree of saturation level (peaks 28, 33 & 36), presented in both pepper samples except the last one found only in white pepper, respective to exact masses of *m*/*z* 293.2117, 295.2272 & 297.2429 (C_18_H_29_O_3_^−^, C_18_H_31_O_3_^−^ & C_18_H_33_O_3_^−^), annotated as hydroxy-octadecatrienoic acid, hydroxy-octadecadienoic & hydroxy-octadecenoic acid, with mass difference 2 amu relative to extra double bond. Moreover, peaks 13 and 16 exceeded peaks 33 & 36 by 32 amu relative to the two extra oxygen atoms of hydroxyl groups. In general, oxylipids were reported for several activities including anti-inflammatory, antimicrobial and cytotoxicity.^52^ The molecular network of fatty acids showing connection between compounds and the mass differences is depicted in (Fig. 5C).

### PCA and OPLS multivariate analyses of black and white pepper samples analysed by UPLC-MS/MS

**Fig. 6:**
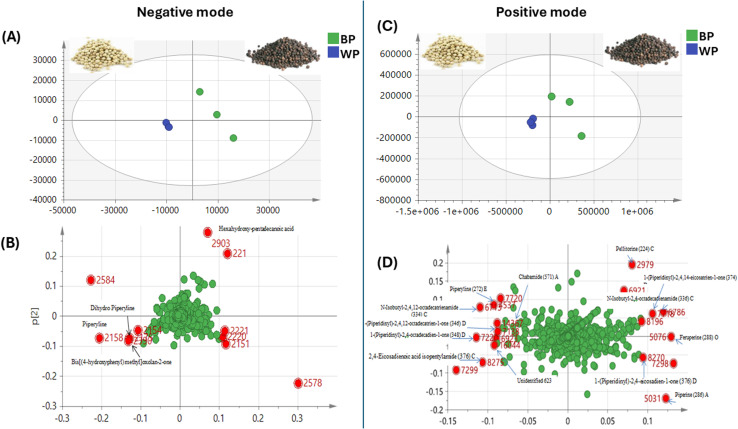
Principal component analysis (PCA) of *Piper nigrum* L. black and white samples analyzed by UPLC-MS/MS (negative and positive modes). (A) PCA score plot negative, (B) loading plot negative with PC1 = 47% and PC2 = 23%. (C) PCA score plot positive (D) loading plots positive with PC1 = 51% and PC2 = 17%.

To better reveal the metabolic difference between black and white *P. nigrum* L. samples, UPLC-MS/MS metabolic profile was modelled using unsupervised PCA analysis. The model accounted for 70% of the total variance that was explained by two components: PC1 (47% & 51%) and PC2 (23% & 17%) in negative (Fig. 6A) and positive modes (Fig. 6C), respectively. Both score plots showed segregation of black and white pepper. Examination of the loading plot in the negative mode (Fig. 6B), revealed that alkaloids detected in negative ion mode *viz*. piperyline and its dihydro derivative with bis[(4-hydroxyphenyl)methyl]oxolan-2-one, were the distinctive metabolites for white pepper. Positive ion mode PCA loading plot (Fig. 6D), revealed different discriminatory piperamides predicted for both samples of varies types, mainly A, C & D (Fig. 6). For example, in positive PC1 region where the black pepper sample was located; distinctive alkaloids; feruperine (type O), *N*-isobutyl-2,4-octadecadienamide (type C), 1-(piperidiny)-2,4-eicosadien-1-one (type D) and 1-(piperidiny)-2,4,14-eicosatrien-1-one (type D). On the other side for the negative PC1 position where white pepper sample was positioned, piperyline (type E), dipiperamide A (type A), 1-(piperidinyl)-2,4-eicosadien-1-one (type D), 1-(piperidinyl)-2,4-octadecadien-1-one (type D), 1-(piperidinyl)-2,4,12-octadecatrien-1-one (type D) and *N*-isobutyl-2,4,12-octadecatrienamide (type C) were the discriminatory piperamides.

**Fig. 7:**
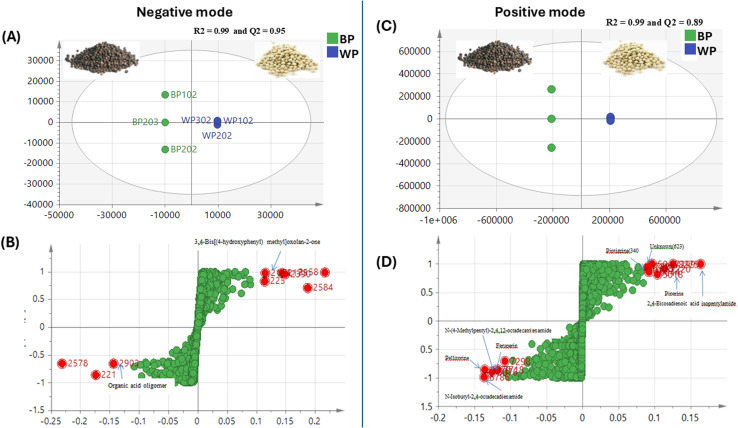
Orthogonal projection to latent structures-discriminant data analysis (OPLS) of *P. nigrum* L. black and white samples analyzed by UPLC-MS/MS (negative and positive modes). (A) Supervised OPLS-DA score plot, (B) loading *S*-plot with *R*^2^ = 0.99 and *Q*^2^ = 0.95. (C) Supervised OPLS-DA score plot and (D) loading *S*-plot with *R*^2^ = 0.99 and *Q*^2^ = 0.89.

OPLS was further employed as a supervised model to confirm results derived from PCA with good variance coverage and prediction power (*R*^2^ = 0.99, 0.99 & *Q*^2^ = 0.95, 0.89) for negative (Fig. 7A) and positive modes (Fig. 7C), respectively. *S*-Loading plots confirmed bis[(4-hydroxyphenyl)methyl]oxolan-2-one as a marker for white pepper in OPLS negative mode (Fig. 7B), while positive mode OPLS (Fig. 7D) revealed, feruperine (type O), *N*-isobutyl-2,4-octadecadienamide (type C) and *N*-(4-methylpentyl)-2,4,12-octadecatrienamide (C) being more enriched in black pepper sample, *versus* 1-(piperidinyl)-2,4-eicosadien-1-one (type D) alongside unknown chemical [M − H]^−^ at *m*/*z* 623 as white pepper discriminatory metabolites.

### Total phenolics and total flavonoids assays in pepper

For preliminary screening, the total phenolic and flavonoid contents of black and white pepper fruit extracts were quantified (Table S2). The results revealed considerable levels of total phenolics 45.6 and 37.8 mg gallic acid equivalents (GAE) per g dry weight for black and white pepper, respectively. In contrast, total flavonoid contents were moderate, at 9.4 and 8.5 mg rutin equivalents (RE) per g dry weight. As expected, black pepper exhibited higher levels of both phenolics and flavonoids compared to white pepper. The aluminium chloride colorimetric assay, used for flavonoid estimation, is selective for specific subclasses such as flavones and flavonols. This specificity may partly explain the observed differences in flavonoid content between the two pepper types.^53^ Our findings are consistent with earlier reports showing total phenolic contents in aqueous and ethanolic black pepper extracts ranging from 42.8 to 54.3 μg GAE per mg, as determined by the Folin–Ciocâlteu method.^54^

### *In vitro* antioxidant assays

To confirm whether the richness of phenolics is reflected in improved antioxidant potential in black pepper, *in vitro* antioxidant assays were estimated for both pepper types using free radical DPPH, ABTS scavenging assays, as well as FRAP reducing assay (Table S2). Results revealed stronger effects in black pepper, as expected, at 49.79 ± 2.01 and 20.57 ± 0.48, *versus* for white pepper, with 29 ± 2.57 and 11.47 ± 0.42 mg TE per g b.wt. for DPPH and ABTS assays. Furthermore, the FRAP assay showed values of 104.56 ± 1.30 and 77.52 ± 2.51 mg TE per g b.wt. for black and white pepper, respectively. The superior antioxidant activity of black pepper may be attributed to its higher phenolic content, which is partly due to the presence of the outer pericarp. During the processing of white pepper, the pericarp is removed, leading to a measurable loss of phenolic compounds known for their antioxidant properties.^55^ This compositional difference likely contributes to the reduced antioxidant potential observed in white pepper. Our findings are in line with previous reports demonstrating the antioxidant and neuroprotective effects of *P. nigrum* extracts, which protect cells against oxidative stress by decreasing ROS production and preserving mitochondrial membrane integrity.^56^ These bioactivities are largely associated with the presence of phenolic and alkaloid constituents retained in the black pepper matrix. Another antioxidative assay of black pepper water and ethanol extracts using 6 different assays such as total antioxidant activity, metal chelating activity, DPPH free radical scavenging, superoxide anion radical scavenging, hydrogen peroxide scavenging, and reducing power, revealed that both extracts are free radical scavenging agents.^54^ Furthermore, the antioxidant potential of black pepper and its constituents was evaluated using the thiobarbituric acid reactive substances (TBARS) assay, which measures the inhibition of lipid peroxidation by detecting malondialdehyde (MDA) levels. The results showed that black pepper extract significantly suppressed MDA formation, indicating strong antioxidant activity.^57^ It could be concluded that piperine, a significant pungent alkaloid, along with flavonoids and phenolics in *P. nigrum* samples contribute to the plant antioxidant action.^58,59^

### *In vitro* enzymes inhibition assays

Inhibitory activity of α-glucosidase and pancreatic lipase (PL) were assessed for black and white pepper extracts (Table S3) to evaluate their antidiabetic and antihyperlipidemic potential, especially being rich in piperine, a major alkaloid that exhibited hypoglycemic action in diabetes induced mice.^60^ Results revealed that black and white pepper fruit extracts inhibited *α*-glucosidase with comparable IC_50_ values of 0.77 ± 0.012 and 0.617 ± 0.138 mg mL^−1^, compared to standard drug, acarbose, showed IC_50_ values of 0.490 mg mL^−1^. In contrast, no significant inhibitory action was recorded against PL inhibitory in comparison to the active drug, orlistat, later showing IC_50_ value of 0.1603 mg mL^−1^. Previous studies in pepper revealed that piperine showed the highest α-amylase & α-glucosidase inhibitory effect compared with the extract.^61^

## Conclusion

Multiplex metabolites profiling approach was employed herein for the assessment of primary and secondary metabolites in black and white pepper samples. Gas chromatography mass-spectrometry (GC-MS) post-silylation and ultra-performance liquid chromatography (UPLC-MS/MS) coupled to multivariate analyses, along with molecular networking, were applied. A total of 51 metabolites were detected by GC-MS post-silylation analysis in black and white pepper samples with the abundance of fatty acids/esters in black and white pepper at a comparable level of *ca.* 23.4 mg g^−1^. Piperine was detected at a higher level in white pepper at 5.9 mg g^−1^ compared to 3.4 mg g^−1^ in black pepper. Moreover, 71 metabolites were annotated using UPLC-MS/MS with an abundance of piperamides with 6 novel alkamides first to be detected in *Piper nigrum* fruit; two of type A, one of type E & three of type O. Furthermore, 4 phenolamides were first identified from pepper, in addition to flavonoid glycosides with novel glycosidic linkage adding to pepper chemical makeup. Neverlessness, future work is recommended for more structural confirmation using NMR. GNPS molecular networking revealed the clusters of fatty acids, flavonoids and phenylamides in negative mode, whereas alkaloids clusters were detected in positive mode MN highlighting the value of dual detection in both modes. Multivariate data analysis revealed distinct variation between black and white pepper samples attributed to piperamides: feruperine (type O), *N*-isobutyl-2,4-octadecadienamide (type C), 1-(piperidiny)-2,4-eicosadien-1-one (type D) and 1-(piperidiny)-2,4,14-eicosatrien-1-one (type D) as black pepper distinctive alkaloids, while piperyline (type E), dipiperamide A (type A), 1-(piperidinyl)-2,4-eicosadien-1-one (type D), 1-(piperidinyl)-2,4-octadecadien-1-one (type D), 1-(piperidinyl)-2,4,12-octadecatrien-1-one (type D) and *N*-isobutyl-2,4,12-octadecatrienamide (type C) were discriminatory piperamides in white pepper. Black pepper extracts were more enriched in phenolics and flavonoids than white pepper likely due to decortication of the outer layer. Moderate antioxidant effect was detected using DPPH, ABTS, and FRAP assays, and yet to be confirmed using *in vivo* animal models. In contrast, potent α-glucosidase inhibition effect was detected with IC_50_ = 0.77 and 0.62 mg mL^−1^ for black and white pepper, respectively, compared to acarbose (0.49 mg mL^−1^). Such promising results present future uses of *P. nigrum* in dietary supplements or nutraceuticals. Owing to a limited sample source from a single geographic origin, future studies incorporating a broader range of samples from different regions and harvest seasons are recommended to validate and expand upon these results using the same analytical platform reported in this study for that chief spice.

## Author contributions

Mohamed A. Farag: supervision, conceptualization, methodology, investigation, writing – review & editing. Amal A. Maamoun: investigation, formal analysis, writing – review & editing. Alexandru Nicolescu: methodology, investigation, writing – review & editing. Andrei Mocan: methodology, investigation, writing – review & editing. Mostafa H. Baky: investigation, formal analysis, writing – review & editing.

## Conflicts of interest

The authors declare no conflicts of interest.

## Supplementary Material

RA-015-D5RA03714J-s001

## Data Availability

All the data regarding our manuscript is available in the main document and its SI. Supplementary information is available: Supplementary Tables S1–S3 and Supplementary Fig. S1–S14. See DOI: https://doi.org/10.1039/d5ra03714j.
