# Supplementary material for: Multi-targeted MS-based metabolomics fingerprinting of black and white pepper coupled with molecular networking in relation to their in vitro antioxidant and antidiabetic effects
Source: RSC Adv. 2025 Aug 4;15(34):27606–22. doi: 10.1039/d5ra03714j (PMC12320482; doi:10.1039/d5ra03714j)
Supplement: RA-015-D5RA03714J-s001 [file RA-015-D5RA03714J-s001.pdf]

Supplementary Information (SI) for RSC Advances.
This journal is © The Royal Society of Chemistry 2025

Supplementary File

Multi-targeted MS-based metabolomics fingerprinting of black and white pepper coupled with molecular networking in relation to their *in vitro* antioxidant and antidiabetic effects

Mostafa H. Baky [1a*], Amal A. Maamoun[2a], Alexandru Nicolescu[3], Andrei Mocan[3,4], Mohamed A. Farag [5]

[1] Department of Pharmacognosy, Faculty of pharmacy, Egyptian Russian University, Badr city, 11829, Cairo, Egypt.

[2]Pharmacognosy Department. National Research Centre. 33 El Buhouth Street. Dokki, Giza, Egypt, (P. O. 12622)

[3]Laboratory of Chromatography, Institute of Advanced Horticulture Research of Transylvania, University of Agricultural Sciences and Veterinary Medicine, 3-5 Mănăștur Street, 400372, Cluj-Napoca, Romania

[4]Department of Pharmaceutical Botany, "Iuliu Hațieganu" University of Medicine and Pharmacy, Gheorghe Marinescu Street 23, 400337, Cluj-Napoca, Romania

[5] Pharmacognosy Department, College of Pharmacy, Cairo University, 11562 Cairo, Egypt.

a: Equal contribution

*Corresponding author at: Cairo University, College of Pharmacy, Department of Pharmacognosy, Cairo, Egypt.

E-mail addresses: mohamed.farag@pharma.cu.edu.eg, (M.A. Farag).

**Table S1.** Major metabolites detected in black and white pepper samples. Values are expressed as mean ± S.D (n = 3). Statistical analysis is carried out by t-Test: Paired Two Sample for Means where significance difference at $p \leq 0.05$ and non-significance at $p > 0.05$.			
**Metabolite**	**BP (mg/g) Mean ± SD**	**WP (mg/g) Mean ± SD**	**t-Test (p-value)**
Oxalic acid (2TMS)	2.44±0.50	2.78±0.17	0.45
γ-Aminobutyric acid, (3TMS) isomer	0.63±0.10	0.06±0.02	0.01
α-Hydroxybehenic acid methyl ester, (TMS)	21.29±21.49	21.06±21.21	0.99
Piperine	3.44±1.45	5.95±0.49	0.13
Glycerol, (3TMS)	0.18±0.02	0.25±0.01	0.07
Myoinositol (TMS)	0.12±0.03	0.02±0.01	0.03

**Table S2.** Results of antioxidant activity, total phenolic and total flavonoids of black and white pepper, values are expressed as mean ± S.D (n = 3). Statistical analysis is carried out by t-Test: Paired Two Sample for Means where significance difference at p < 0.05.				
**Sample ID**	**BP**	**WP**		**p-value (t-Test)**
DPPH	52.0692	31.7324		
	48.2317	26.6145		
	49.0845	28.6617		
avg	**49.7951***	**29.0028***	**mgTE/g w**	0.0004
stdev ±	2.01507	2.57594		
ABTS	21.088	11.386		
	20.1344	11.1057		
	20.4977	11.9467		
avg	**20.5733***	**11.4795***	**mgTE/g w**	0.001
stdev ±	0.4813	0.42818		
FRAP	104.806	74.6267		
	105.725	79.1665		
	103.151	78.7881		
avg	**104.561***	**77.5271***	**mgTE/g w**	0.004
stdev ±	1.30443	2.51895		
TPC Folin	44.8976	35.2252		
	44.3379	37.4737		
	47.5094	39.7221		
avg	**45.5816***	**37.4737***	**mgGAE/g w**	0.01
stdev ±	1.6928	2.24847		
TFC AlCl3	10.0482	7.71191		
	8.76865	7.60223		
	9.4084	10.081		
avg	**9.4084****	**8.46504****	**mgRE/g w**	0.39
stdev ±	0.63975	1.40052		
* p < 0.05<br>** p > 0.05				

**Table S3.** Enzyme inhibition activity of black and white pepper, values are expressed as mean ± S.D (n = 3). Statistical analysis is carried out by t-Test: Paired Two Sample for Means where significance difference at $p < 0.05$ and non-significance at $p > 0.05$.							
**Sample ID**		**IC50 (mg/mL) - replicates**			**Final**	±	**SD**
		**1**	**2**	**3**			
BP	**Alpha-glucosidase**	0.7858	0.7637	0.7688	**0.7726****	±	**0.012**
WP		0.7213	0.4654	0.6821	**0.6179****	±	**0.138**
Acarbose		**0.4891**					
BP	**Lipase**	NA	NA	NA	NA	±	
WP		NA	NA	NA	NA	±	
** $p > 0.05$							

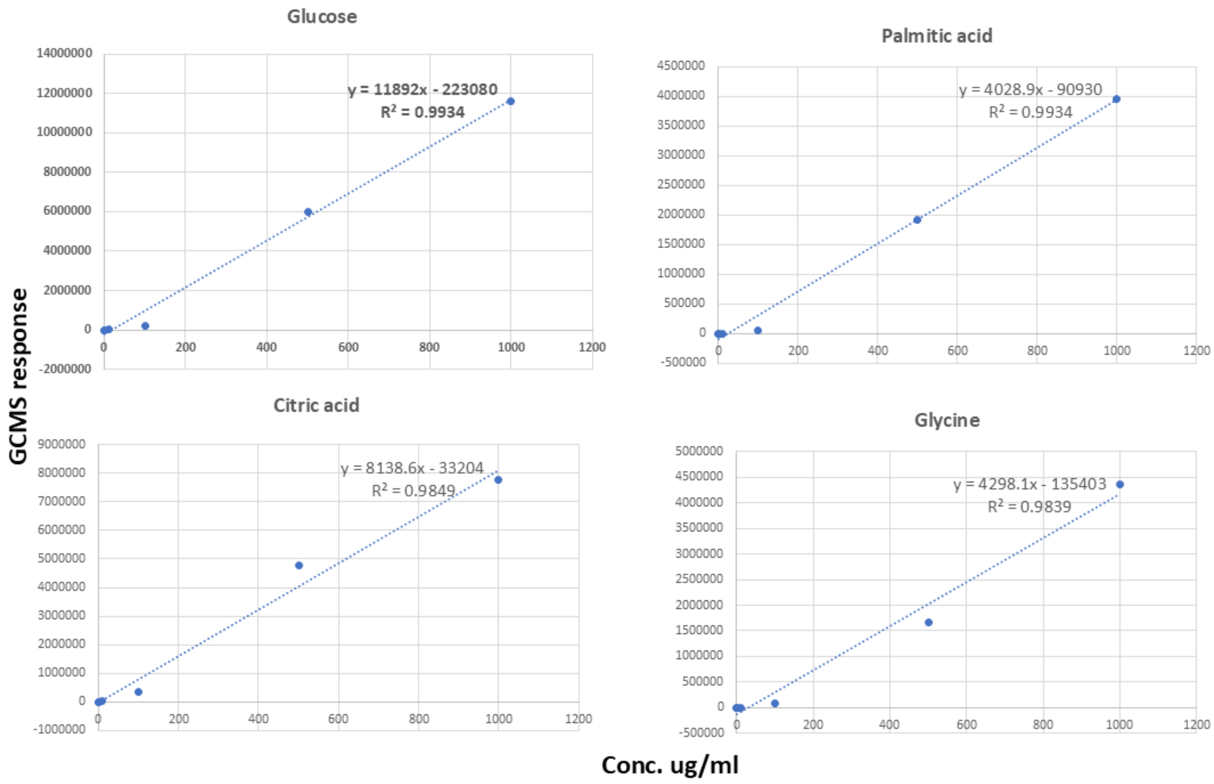

Figure S1. Calibration curves for glucose, glycine, citric acid and palmitic acid displayed 0.9948 correlation coefficient.

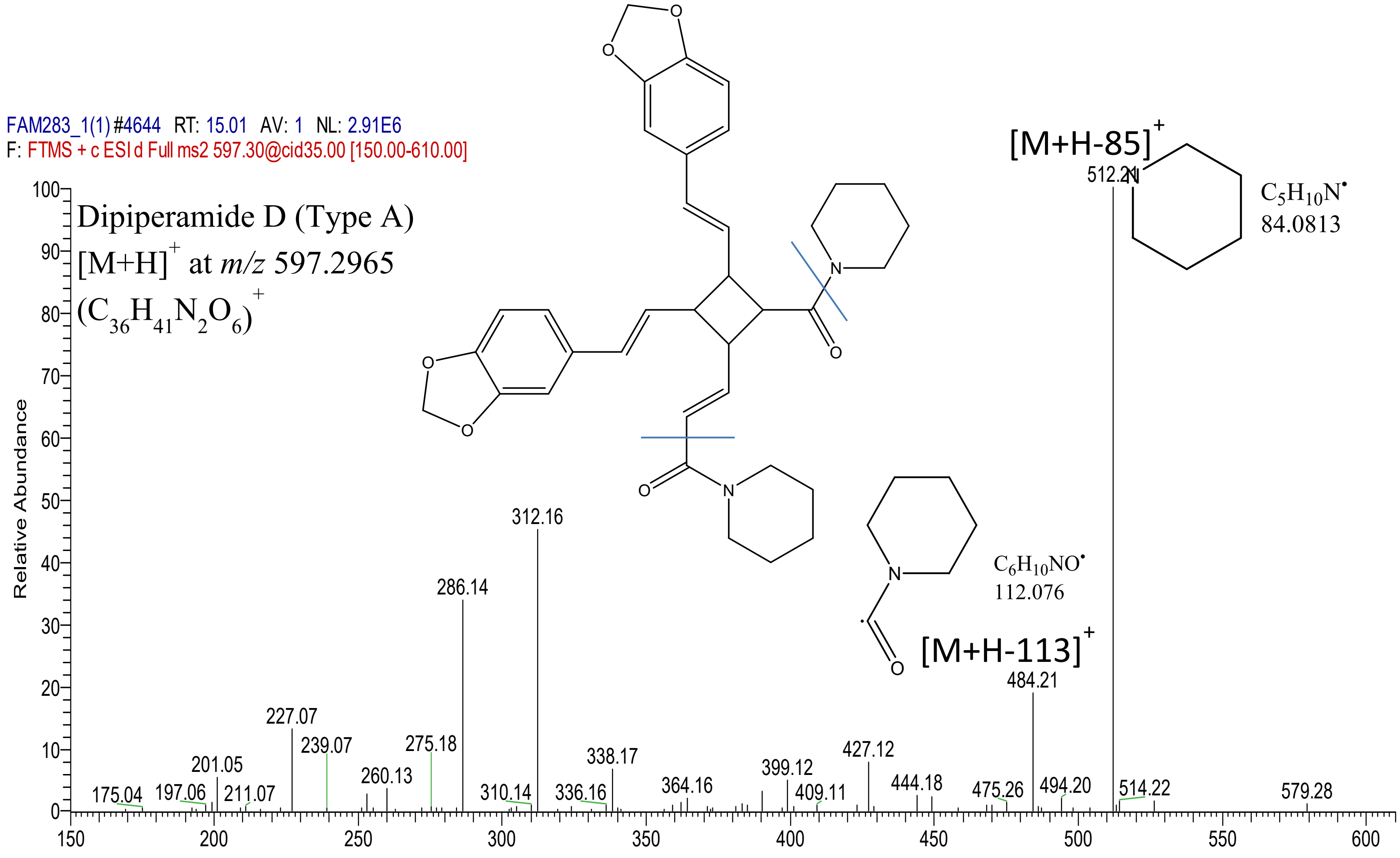

Figure S2. MS_2 spectrumof dipiperamide D (Type A piperamide).

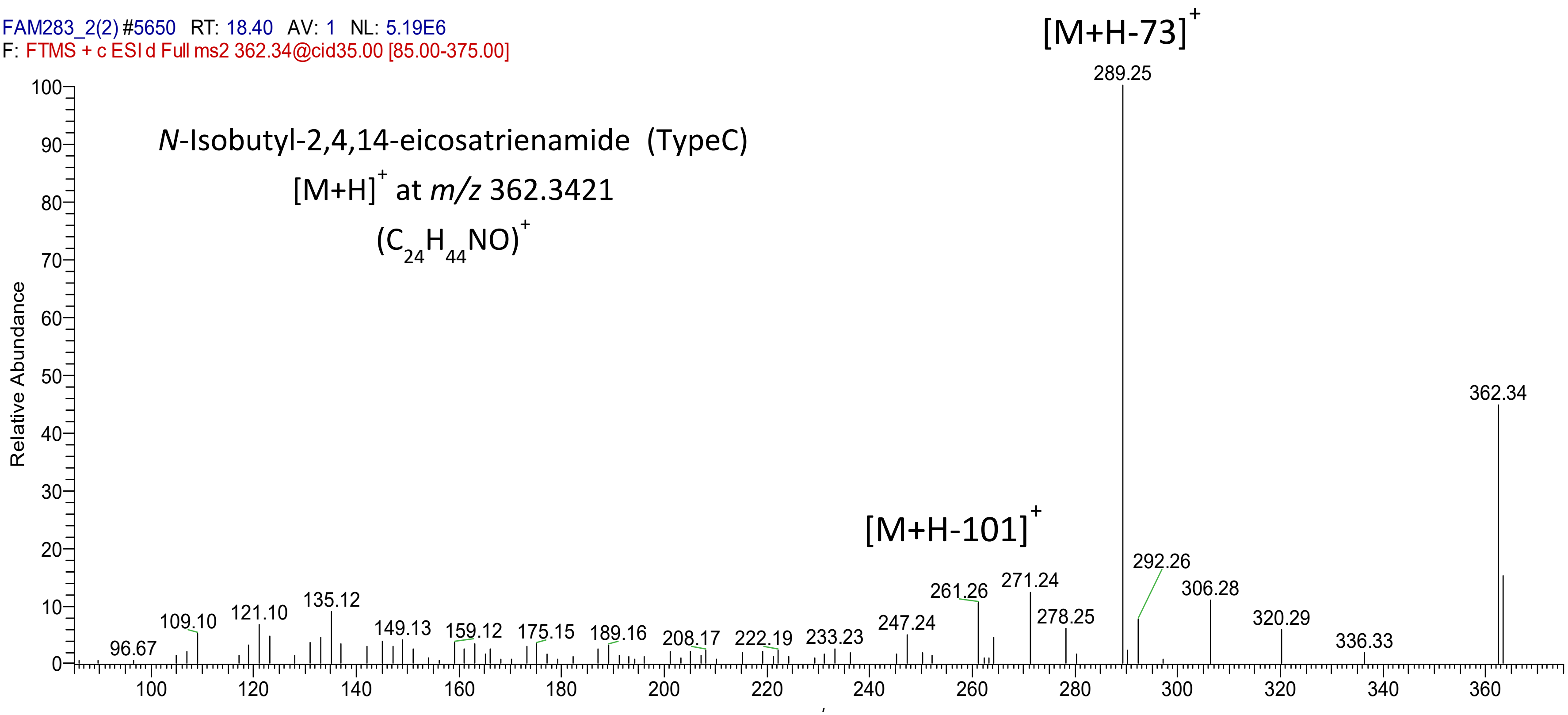

Figure S3. MS_2 spectrumof *N*-isobutyl-2,4,14-eicosatrienamide (Type C piperamide).

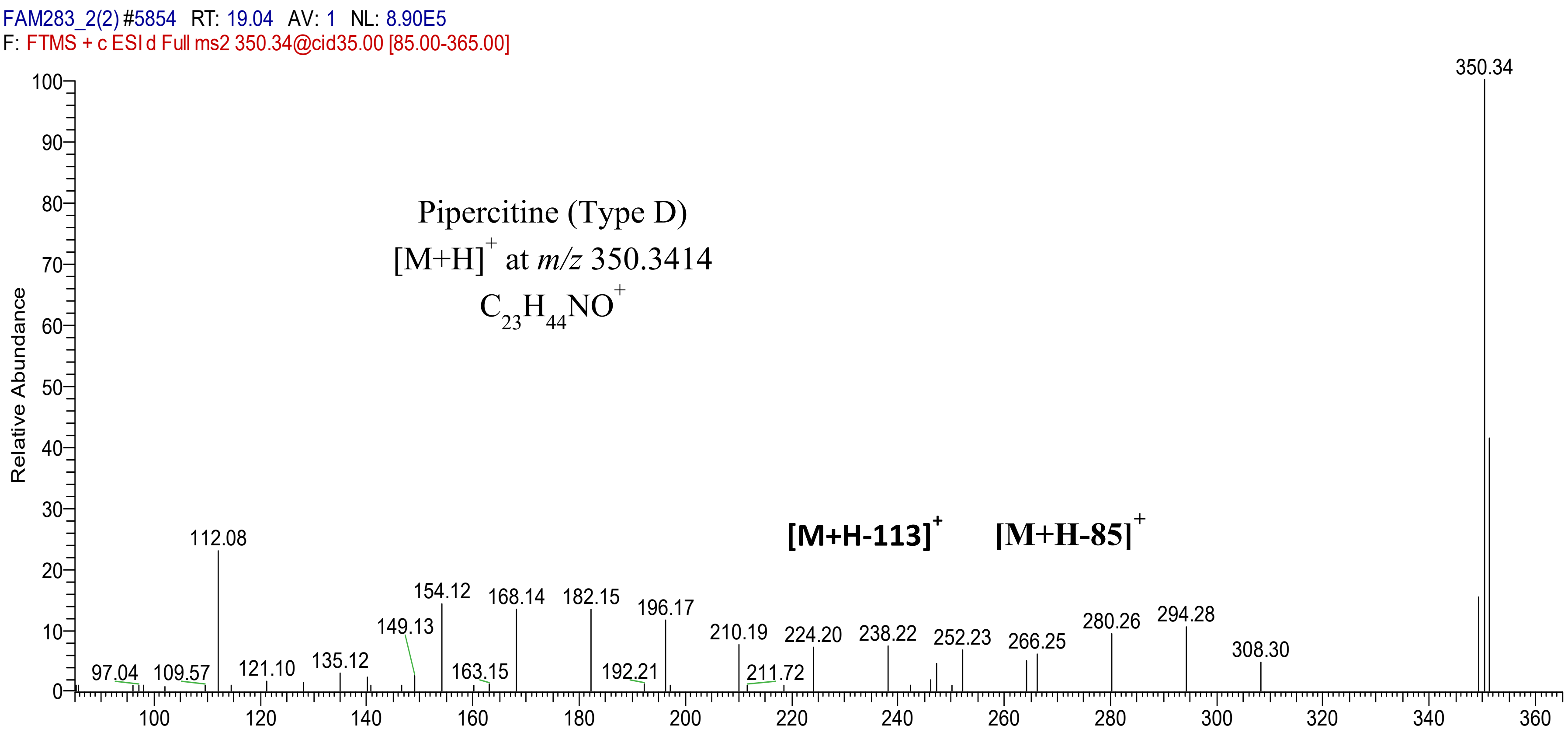

Figure S4. MS_2 spectrumof pipercitine (Type D piperamide).

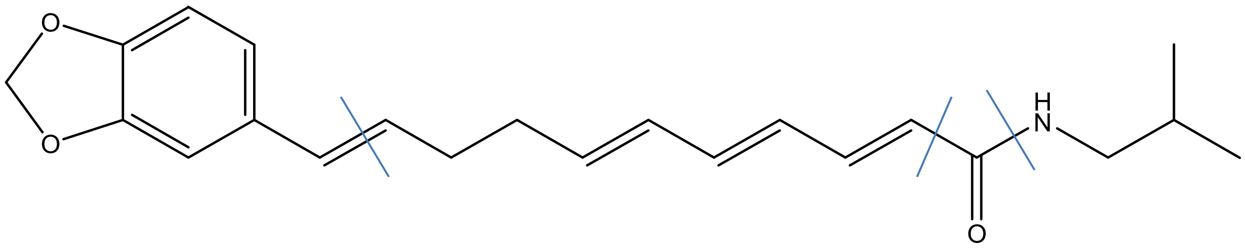

(2E,4E,6E,10E)-11-(1,3-benzodioxol-5-yl)-N-(2-methylpropyl)undeca-2,4,6,10-tetraenamide

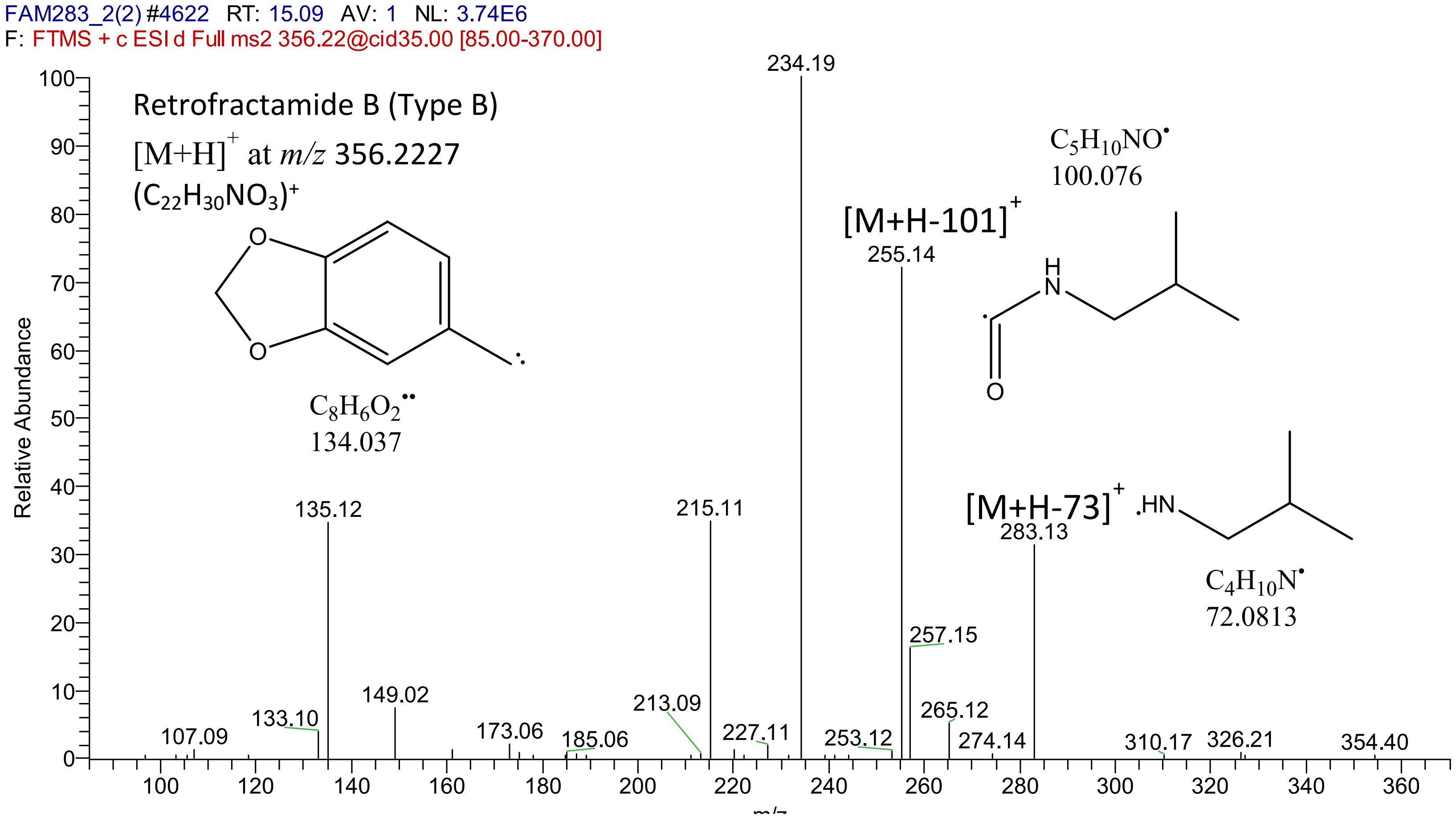

Figure S5. MS_2 spectrum of retrofractamide B (Type B piperamide).

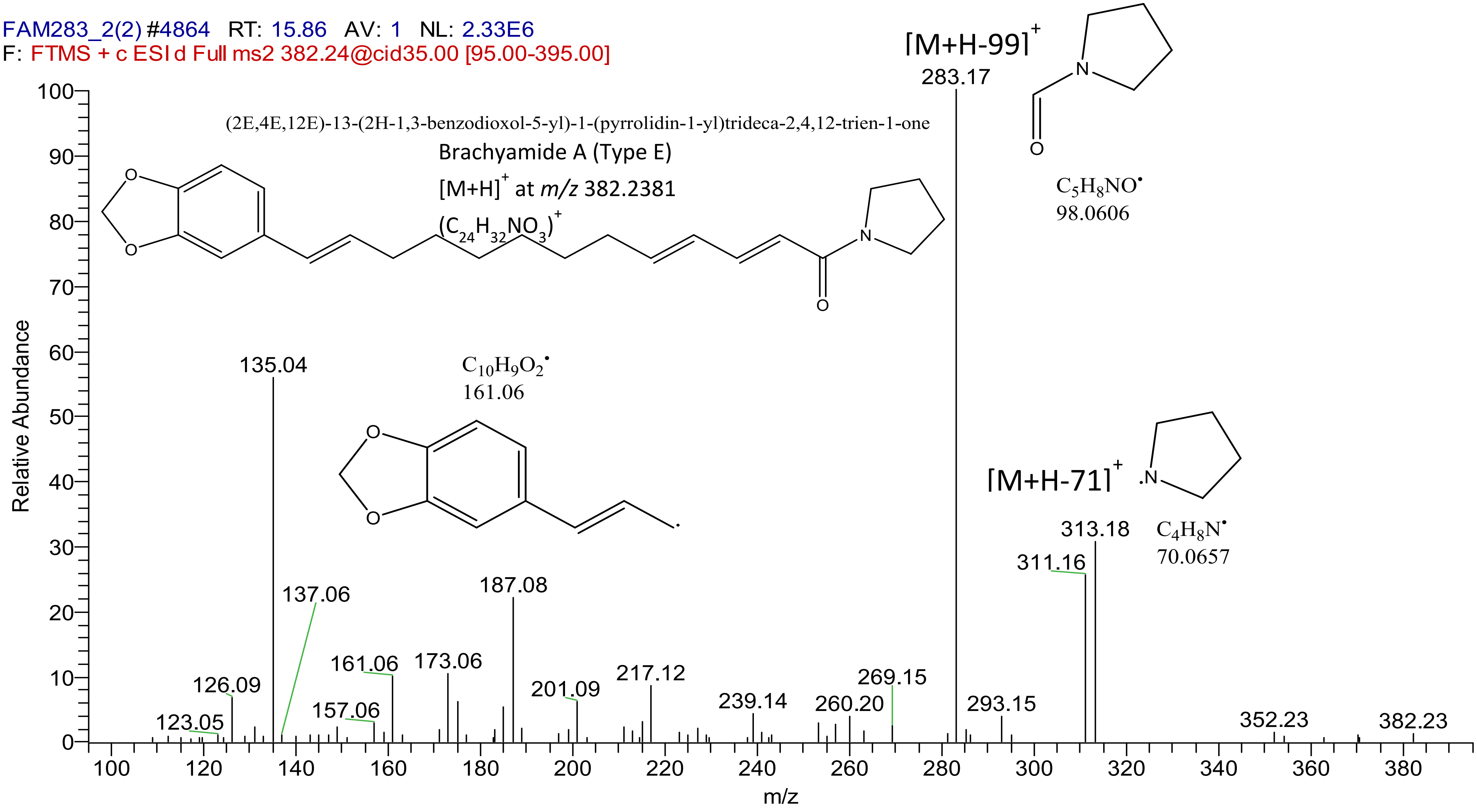

Figure S6. MS_2 spectrum of brachyamide A (Type E piperamide).

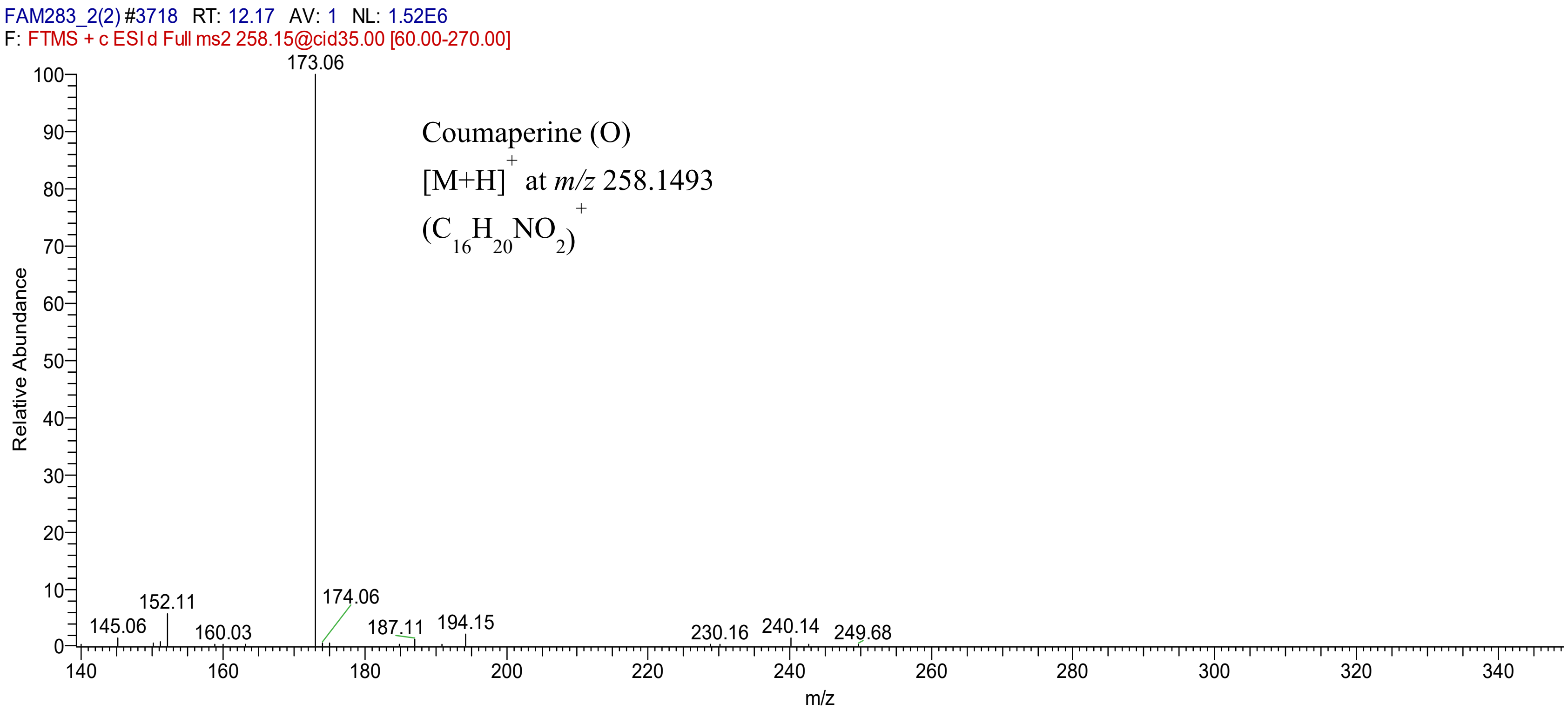

Figure S7. MS_2 spectrum of coumaperine (Type O piperamide).

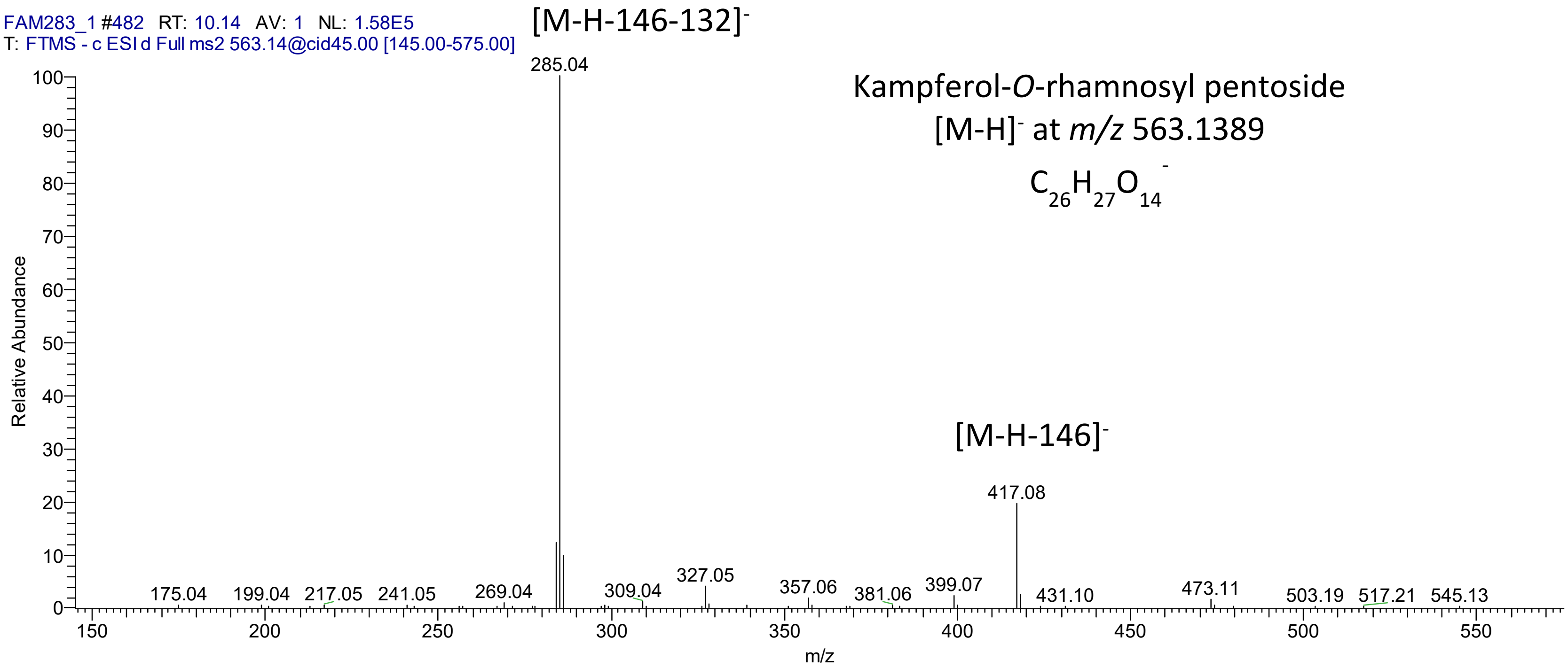

Figure S8. MS_2 spectrum of kampferol-*O*-rhamnosyl pentoside (flavonoid)

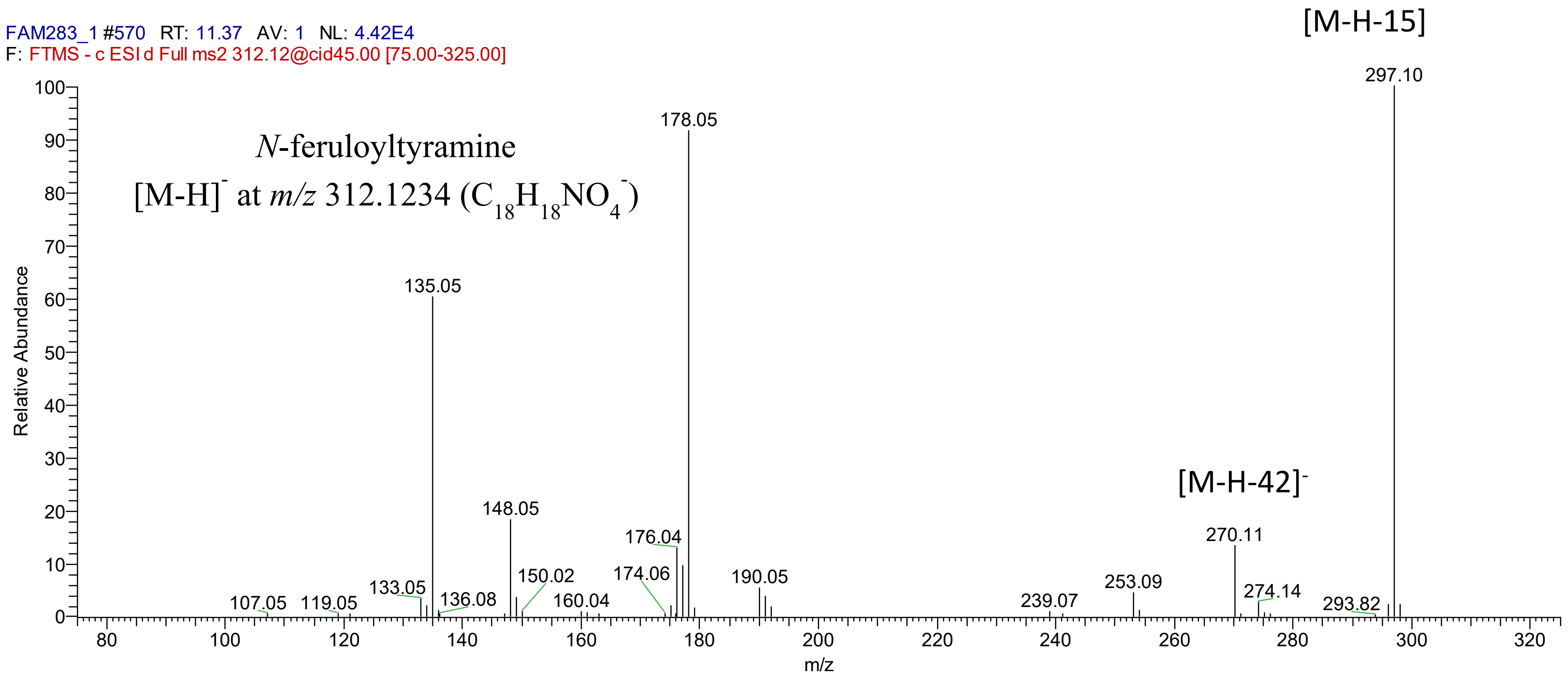

Figure S9. MS_2 spectrum of 3,4-Bis[(4-hydroxyphenyl) methyl]oxolan-2-one *N*-feruloyltyramine (phenylamide)

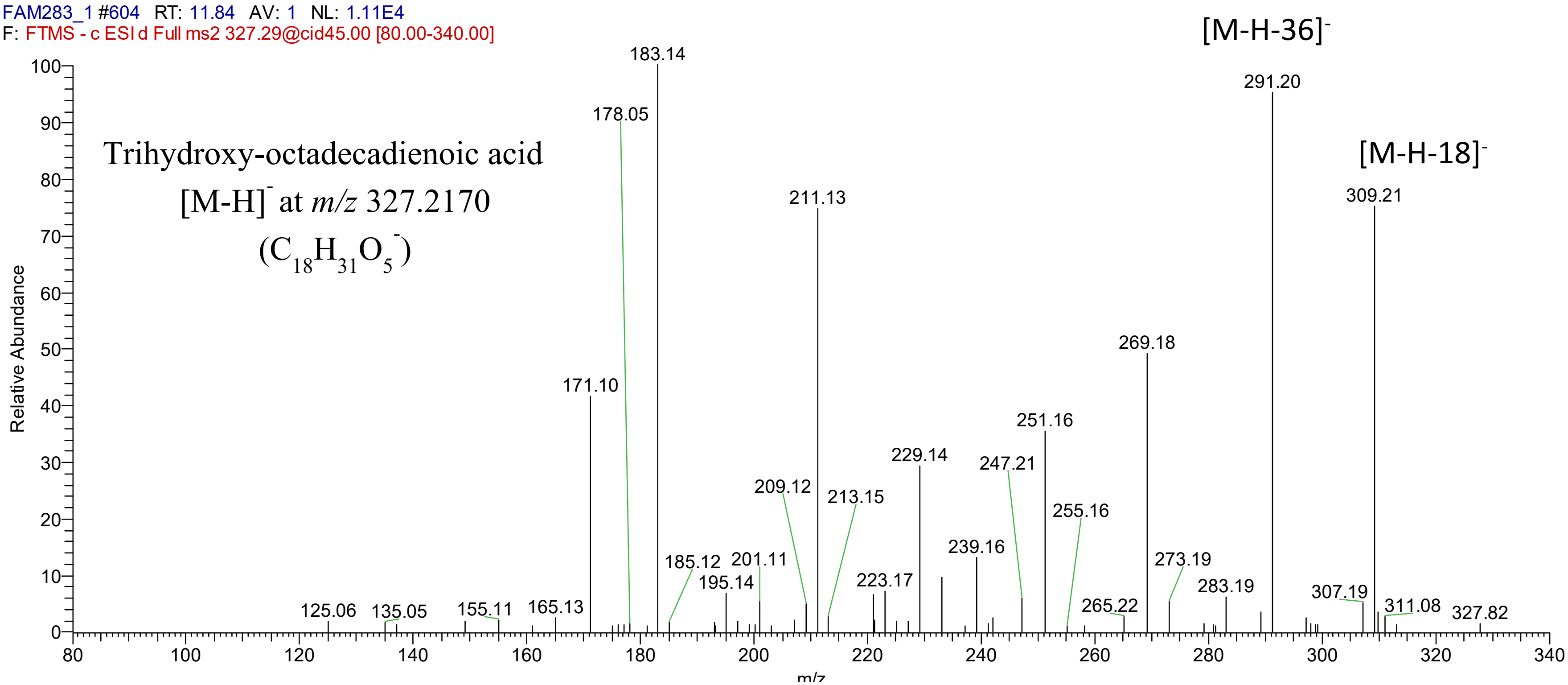

Figure S10. MS_2 spectrum of trihydroxy-octadecadienoic acid (fatty acid)

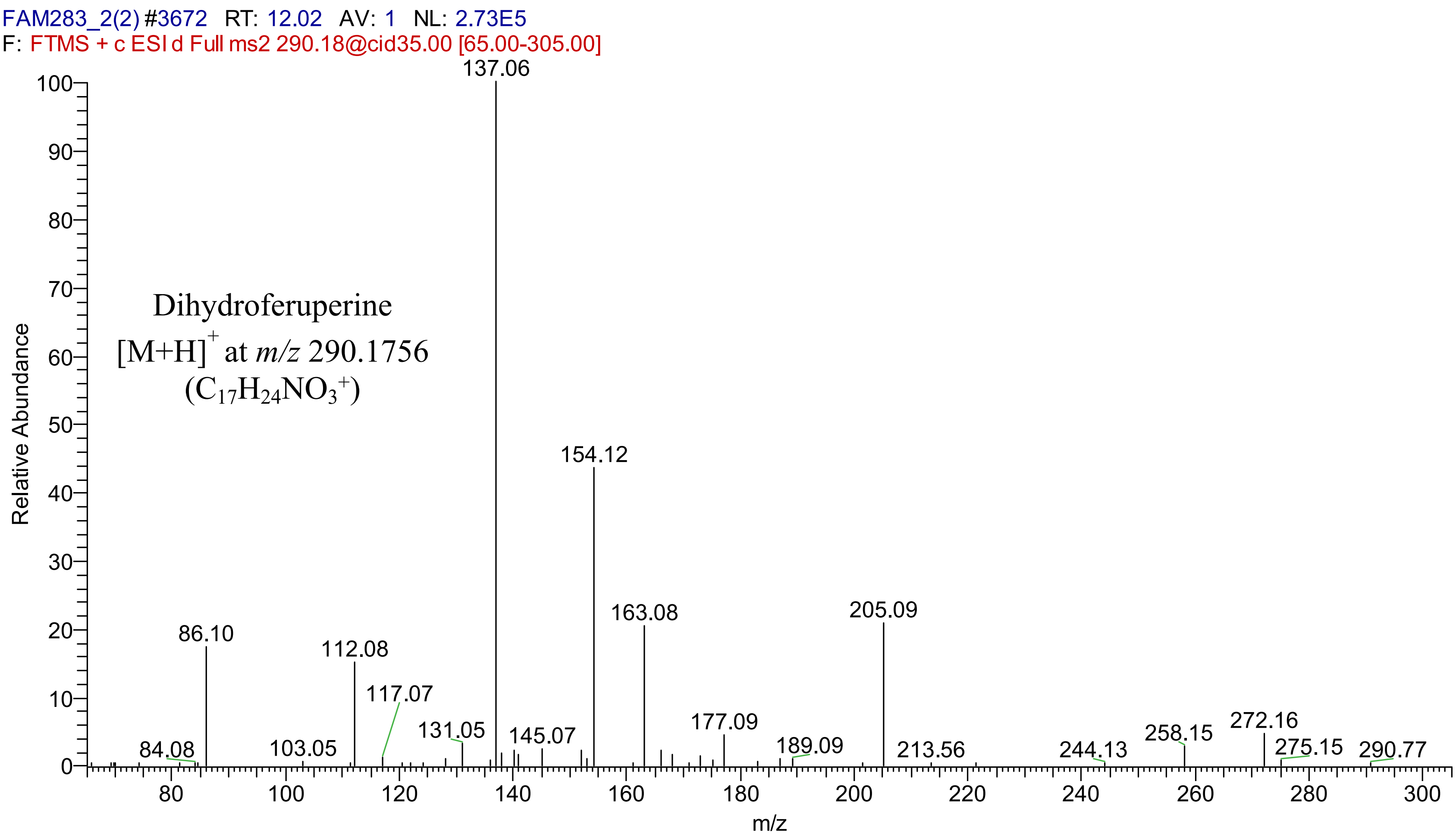

Figure S11. MS_2 spectrum of dihydroferuperine (Type O piperamide)

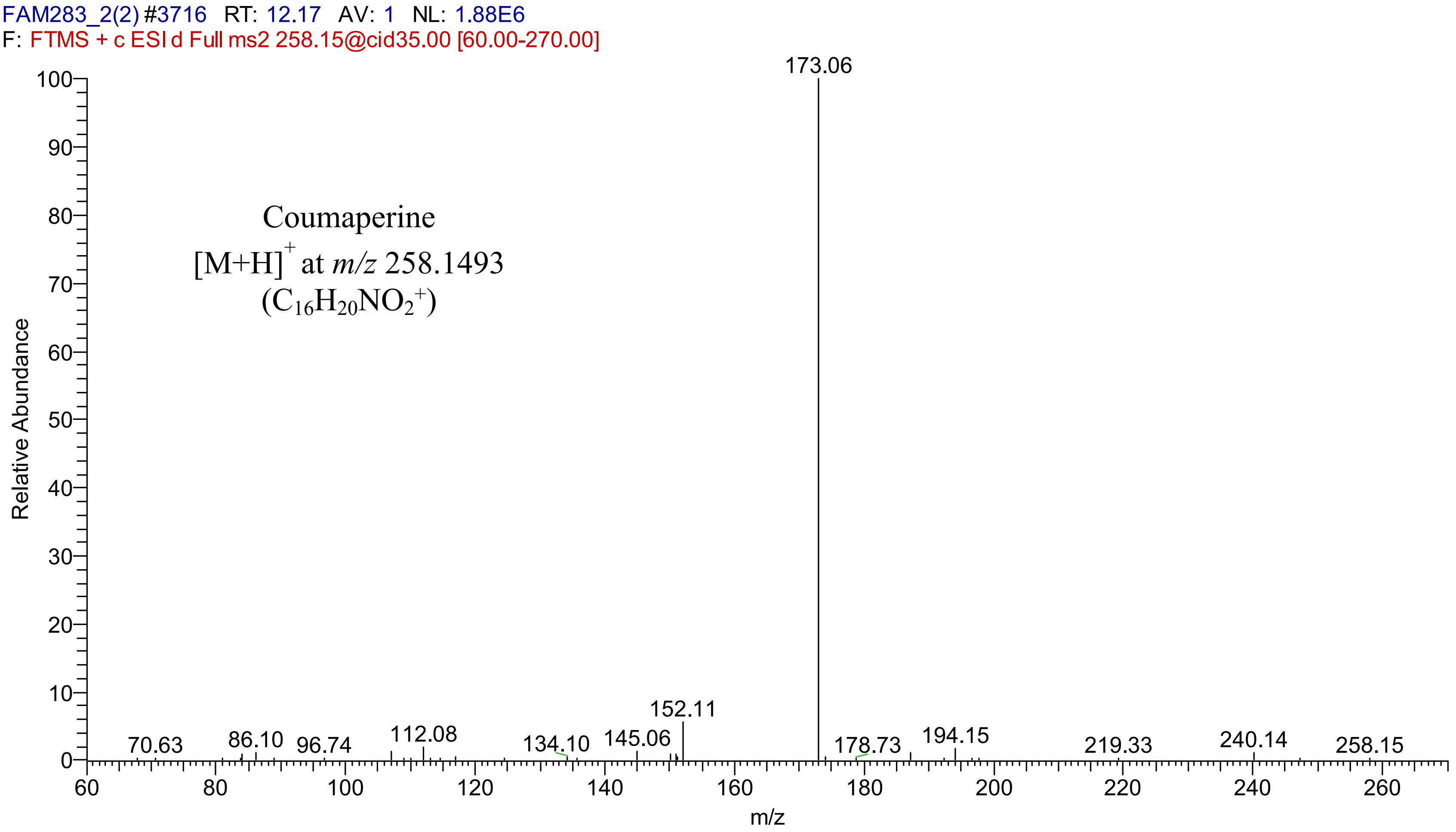

Figure S12. MS_2 spectrum of coumaperine (Type O piperamide)

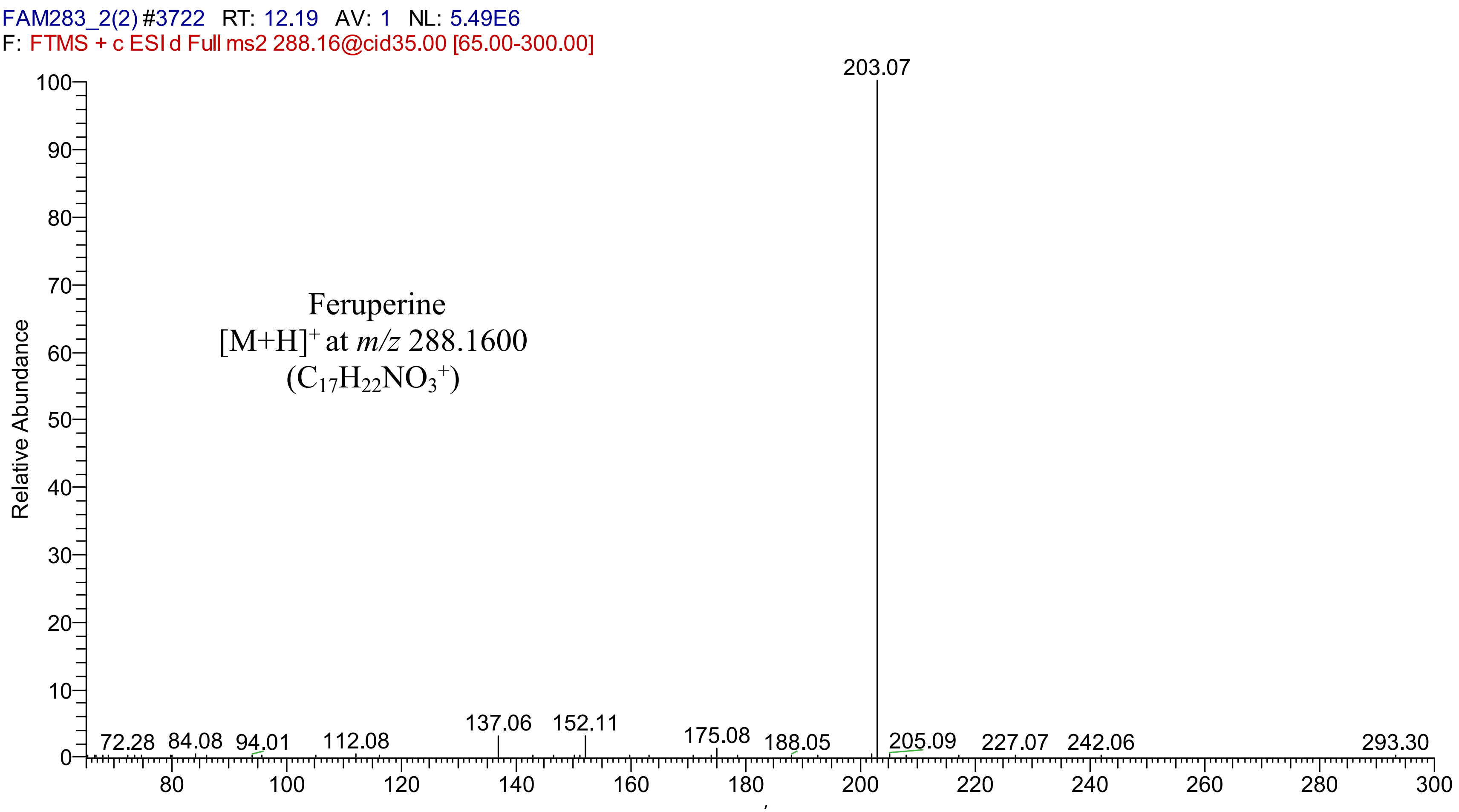

Figure S13. MS_2 spectrum of Feruperine (Type O piperamide)

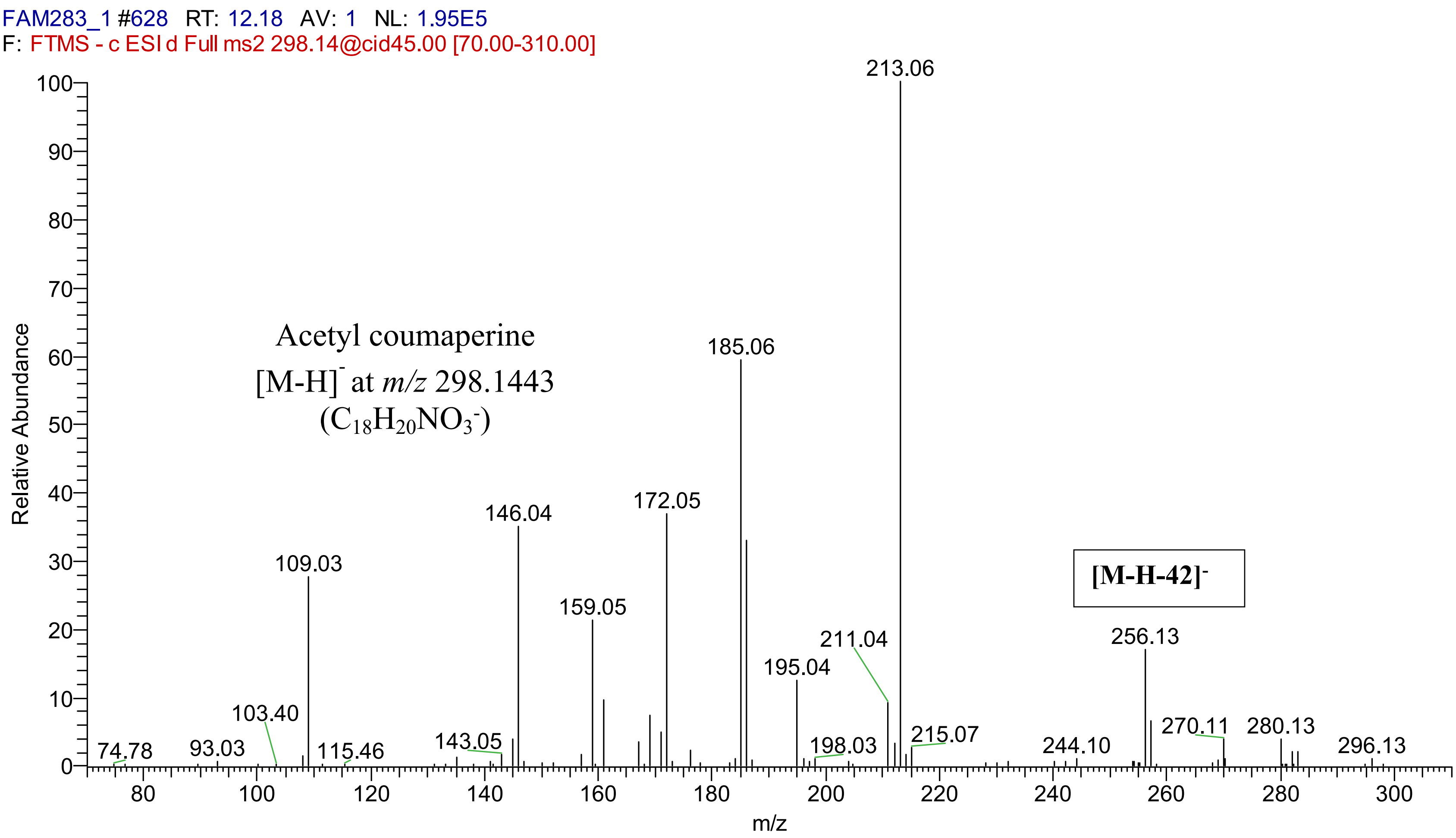

Figure S14. MS_2 spectrum of acetyl coumaperine (Type O piperamide)
